# Curcumin, an active component of turmeric: biological activities, nutritional aspects, immunological, bioavailability, and human health benefits - a comprehensive review

**DOI:** 10.3389/fimmu.2025.1603018

**Published:** 2025-08-21

**Authors:** Mohamed T. El-Saadony, Ahmed M. Saad, Dina Mostafa Mohammed, Samar Sami Alkafaas, Soumya Ghosh, Shaimaa H. Negm, Heba M. Salem, Mohamed A. Fahmy, Walid F. A. Mosa, Essam H. Ibrahim, Synan F. AbuQamar, Khaled A. El-Tarabily

**Affiliations:** ^1^ Department of Agricultural Microbiology, Faculty of Agriculture, Zagazig University, Zagazig, Egypt; ^2^ Department of Biochemistry, Faculty of Agriculture, Zagazig University, Zagazig, Egypt; ^3^ Nutrition and Food Sciences Department, National Research Center, Dokki, Giza, Egypt; ^4^ Molecular Cell Biology Unit, Division of Biochemistry, Department of Chemistry, Faculty of Science, Tanta University, Tanta, Egypt; ^5^ Natural and Medical Sciences Research Center, University of Nizwa, Nizwa, Oman; ^6^ Department of Home Economics, Specific Education Faculty, Port Said University, Port Said, Egypt; ^7^ Department of Poultry Diseases, Faculty of Veterinary Medicine, Cairo University, Giza, Egypt; ^8^ Plant Production Department (Horticulture-Pomology), Faculty of Agriculture, Saba Basha, Alexandria University, Alexandria, Egypt; ^9^ Biology Department, Faculty of Science, King Khalid University, Abha, Saudi Arabia; ^10^ Department of Biology, College of Science, United Arab Emirates University, Al Ain, United Arab Emirates

**Keywords:** bioavailability, *Curcuma longa*, medicinal properties, pharmacological actions, traditional medicine, turmeric

## Abstract

Curcumin (1,7-bis-(4-hydroxy-3-methoxyphenyl)-hepta-1,6-diene-3,5-dione) is a naturally occurring polyphenol molecule. It is lipophilic and has demonstrated *in vitro* and *in vivo* therapeutic effects through multiple pathways. Extensive studies on its pharmacological properties have shown its anti-inflammatory, antioxidant, antinociceptive, antimicrobial, antiparasitic, antimalarial, and wound-healing properties. However, its limited bioavailability in humans due to poor intestinal absorption, rapid metabolism, and rapid systemic elimination remains a significant challenge. Various curcumin formulations have been developed to address this limitation. This article reviews current studies on the biological and pharmacological properties of curcumin. It also examines methods for curcumin isolation, including pressurized fluid extraction, Soxhlet extraction, enzyme-assisted extraction, and microwave extraction. Furthermore, analytical methods for the identification and quantification of curcumin in diverse matrices, as well as procedures for formulating curcumin, will also be addressed. This review consolidates recent studies on curcumin’s chemical, bioactive, and pharmacological properties. It also highlights significant knowledge gaps, indicating the need for future research to elucidate curcumin’s mechanism of action, safety, efficacy, and therapeutic potential for treating various human and animal diseases.

## 1 Introduction

Traditional medicine practitioners are sources of primary healthcare in many low-income countries. According to the World Health Organization, traditional medicine constitutes the primary healthcare system for over 80% of the world’s population (1). People in industrialized nations are increasingly turning to natural remedies, especially herbal ones, as they are considered safer alternatives to traditional drugs (2). However, the process of discovering new medications from natural sources is a complex and costly endeavor. It involves numerous steps, including gathering plant material, extracting active compounds, isolating and purifying these compounds, and finally, characterizing their properties. The final step of this process is the evaluation of its pharmacological and toxicological properties. Despite these challenges, natural products remain a rich source of compounds with unique chemical structures and mechanisms of action, making them potential candidates for treating various human disorders (3).

Turmeric (*Curcuma longa* L.) has been extensively researched, and its use is well-documented in the history of Asian traditional medicine. This includes practices from Austronesian peoples with animistic traditions, Siddha, Traditional Chinese Medicine, Unani Medicine, and Ayurveda (4). The safety of turmeric is evidenced by its staple dietary use across various cultures for centuries. Furthermore, it has also been used for managing several disorders, including diabetes, Alzheimer’s disease, cancer, and rheumatic disorders (5). Turmeric supplementation is linked to multiple health benefits, which include its anti-inflammatory and antioxidant properties (6).

Turmeric is a widely used spice available globally, especially in the Indian subcontinent (7, 8). Its rhizomes can be consumed fresh, cooked, dried, and ground into rich orange-yellow powder. The rhizome’s intense yellow color makes it a natural food coloring additive (9). It is also a seasoning agent in Asian cuisine, particularly in curries, and serves as a dye (8, 10, 11). Turmeric powder has an earthy, mustard-like aroma with a hint of black pepper. The highest diversity of *Curcuma* species is found in Thailand and India. However, many wild species exist in other tropical Asian countries (12, 13).

**Figure 1:**
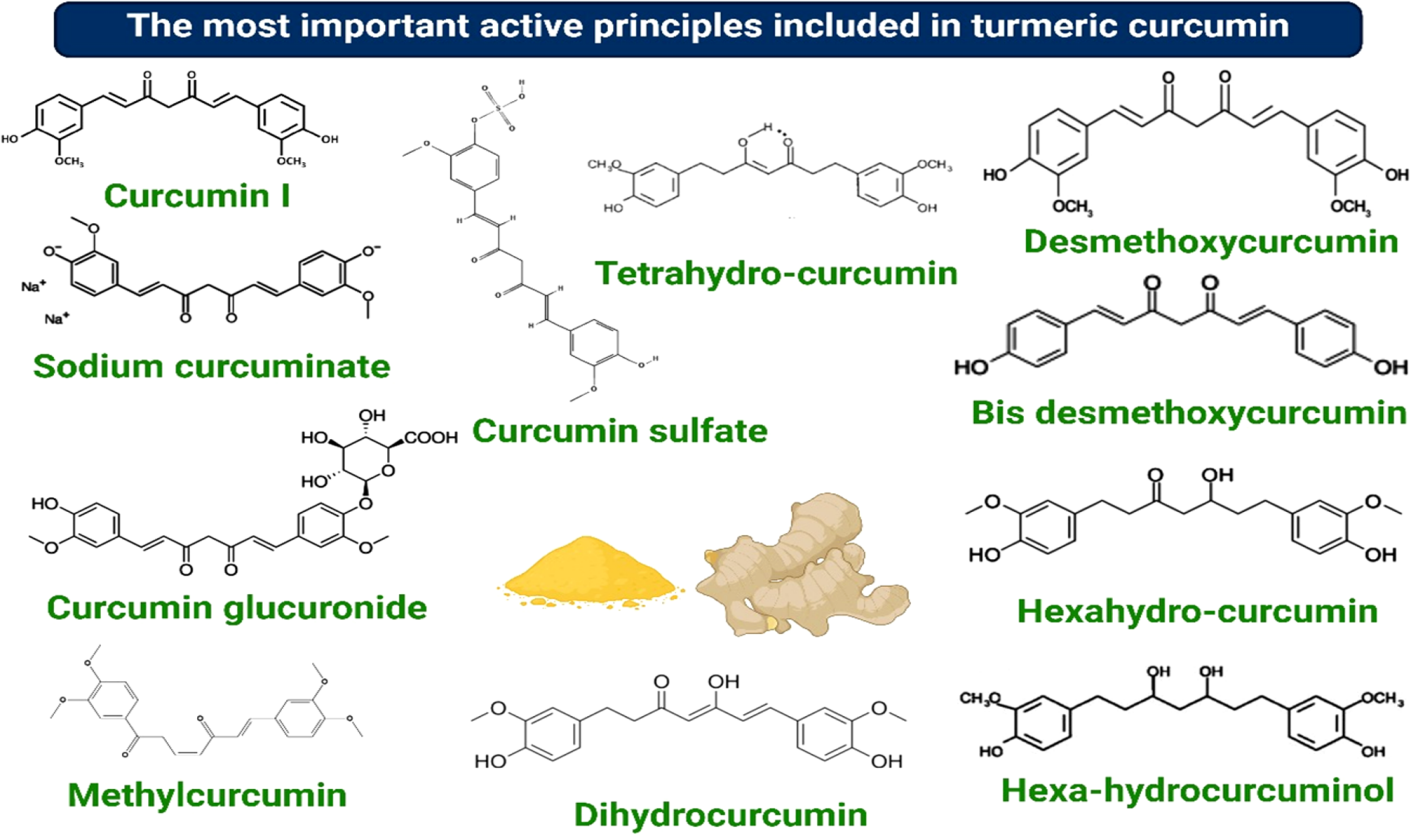
The main active ingredients present in curcumin.

Recent studies have highlighted challenges in classifying *Curcuma* species, with specimens from South India primarily identified as *C. longa*. However, the phylogeny, the intra- and interspecific variation, and the relationships among other *Curcuma* species and cultivars across different regions remain unclear (14). Several species marketed globally as turmeric have been shown to belong to distinct but morphologically similar groups, often sharing comparable local names. *C. longa* grows similarly to ginger and produces rhizomes that contain curcuminoids, including curcumin, desmethoxycurcumin, and bisdemethoxycurcumin (**Figure 1**).

Curcumin is a low-molecular-weight, lipophilic polyphenol that can easily cross cell membranes (9, 15). Curcumin interacts with various cellular signaling pathways, enabling it to modulate some chronic illnesses by binding to key molecules (16), such as transcription factors, inflammatory mediators, and enzymes like protein kinase, reductase, and histone acetyltransferase. It effectively regulates epigenetic modifications associated with neurological diseases, inflammation, diabetes, and various cancers (17). Additionally, curcumin selectively inhibits phosphorylase kinase, reducing glycogen metabolism and altering proteasomal pathways (18).

Curcumin has been widely researched for its diverse health benefits, including anti-inflammatory, antidiabetic, neuroprotective, and disease-fighting properties (19). Despite these promising effects, curcumin’s therapeutic potential is significantly limited by its low bioavailability and poor water solubility (20, 21). Its rapid metabolism, limited absorption, and swift systemic elimination further reduce its concentration in blood plasma and tissues, restricting its clinical effectiveness (22). To address these challenges, it is essential to improve curcumin’s physicochemical properties, particularly its solubility and bioavailability.

Employing advanced formulation techniques is crucial for ensuring the safe and effective therapeutic use of curcumin (22, 23). Recent innovations such as phospholipid complexes, nanoparticles, micelles, hydrogels, and liposomes have demonstrated enhanced efficacy and safety profiles, offering new hope for maximizing the clinical benefits of curcumin (21, 23).

**Figure 2:**
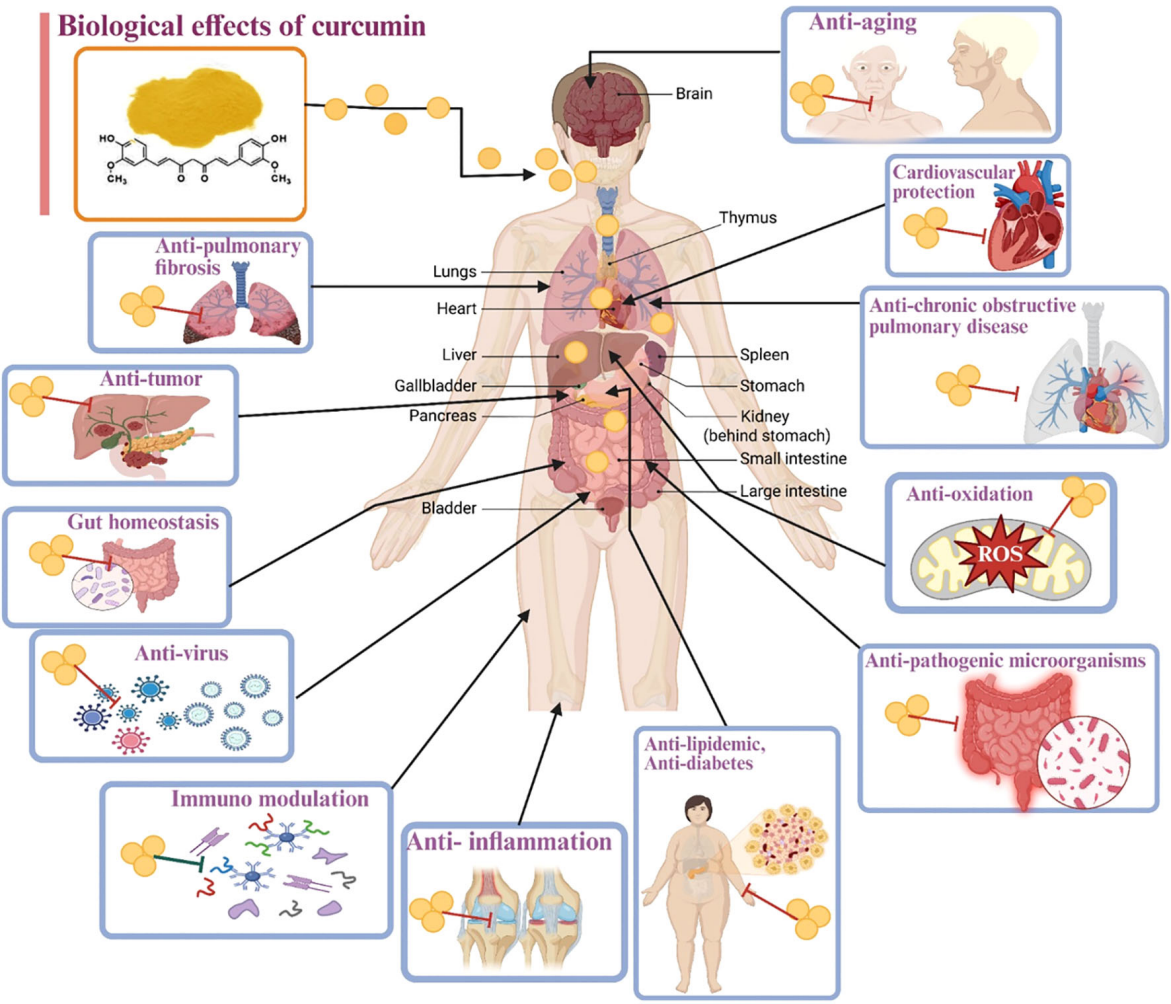
Biological effects of curcumin.

This review distinguishes itself from existing literature by offering a unique and comprehensive synthesis of curcumin research. While prior reviews have covered curcumin’s chemical composition and biological activities, this work goes further by integrating the latest advancements in several key areas. Specifically, it provides an updated understanding of curcumin’s precise immunomodulatory mechanisms, detailing how it influences immune cell function and cytokine pathways, which is crucial for its therapeutic applications in inflammatory and immune-mediated diseases. Furthermore, this review incorporates novel applications, such as its role in managing diseases like COVID-19, including its antiviral properties and ability to enhance vaccine responses, an area of critical recent interest that has not been extensively covered in earlier reviews. **Figure 2** displays the different biological effects of curcumin on human health.

Crucially, this review offers a detailed examination of current and emerging strategies to enhance curcumin’s notoriously low bioavailability. It moves beyond traditional methods to discuss novel approaches such as Self-microemulsifying drug delivery systems (SMEDDS), prodrugs, co-crystallization, and amorphous solid dispersions, providing practical insights for researchers and clinicians working to overcome this significant hurdle. By highlighting both the complex synthesis challenges and the latest solutions for enhancing its pharmaceutical properties, this review offers a more comprehensive and forward-looking perspective on curcumin’s evolution from traditional medicine to modern therapeutics.

This comprehensive update on bioavailability strategies, coupled with insights into novel immunological and antiviral roles, offers significant added value compared to existing reviews, addressing the pressing need for effective and safe translation of curcumin into clinical practice.

## 2 Global distribution of *C. longa*

*C. longa* is a rhizome of the family Zingiberaceae primarily cultivated in southwestern and southern Asia (24). It is widely used in traditional Asian cuisine and is colloquially called the “golden spice” because of its intense golden/yellow color and distinct flavor (24). Apart from its use as a condiment, it impacts on the color and flavor of food. Turmeric powder, derived from its rhizome, is a popular spice and the main ingredient in curry. The powder is also utilized globally as a food coloring and, more recently, as a dietary supplement (24).

Curcumin, a yellow-orange pigment extracted from turmeric, is a natural dye recognized as Natural Yellow 3 (E100) and considered eco-friendly (25, 26). Turmeric is cultivated extensively in many countries, particularly in South Asia, Southeast Asia, and the Middle East. India is the largest producer and consumer of turmeric. Other significant producers include Bangladesh, Pakistan, Sri Lanka, and Indonesia, with countries like China, Japan, Korea, and Australia also growing turmeric (27). It has gained significant popularity in Western countries due to its reported health benefits, resulting in a rise in global demand (27).

## 3 Botanical description of *C. longa*

Turmeric is a herbaceous perennial plant that grows up to 1 m tall. Its rhizomes are cylindrical and branched, with a pleasant aroma and range in color from bright yellow to orange (12). The plant has two alternating rows of leaves, each comprising three parts: the petiole, blade, and sheath. The leaf sheaths form a pseudostem, while the petioles measure from 50 to 115 cm in length (12, 14). Leaf blades typically range between 76 and 115 cm long, with some reaching up to 230 cm, and are 38–45 cm wide (12, 14, 28, 29). Their shape varies from oblong to elliptical, tapering towards the tip (28).

The stem bracts exhibit colors ranging from white to green, often tinged with reddish-purple hues near the inflorescence apex. The bracts taper towards their apices (29). Turmeric flowers are hermaphroditic, trimerous, and zygomorphic. The three united sepals are white, pubescent, and irregularly toothed (28, 29). The flowers have three triangular petals measuring between 1 and 1.5 cm, with soft, spiny protrusions at their tips (12, 14, 28, 29). The corolla tube, formed by the fusion of three bright yellow petals, measures approximately 3 cm in length (29).

## 4 Morphology of *C. longa*

The anther base is equipped with spurs, and staminodes are formed from the residual stamens, with the inner and outer staminodes differing in length (29). The yellow, oval-shaped labellum is between 1.2 and 2.0 cm long and has a yellow ribbon-like marking at its center (14, 29). The bracts are pale green, 3–5 cm long, elliptical to oblong, with a blunt apex (14, 29).

The fruit capsule has three visible sections when opened. In East Asia, the flowering season usually begins in August (29). During this season, an inflorescence stalk, 12–20 cm long and covered with multiple blossoms, develops at the end of the pseudostem (14, 29).

## 5 Chemical composition of *C. longa*

*C. longa* contains various bioactive components, including polysaccharides, essential oils, and curcuminoids (30). It is known for its potential biological and therapeutic effects (31), largely attributed to the quality and concentration of these bioactive components (32). Curcuminoids are polyphenolic compounds responsible for the yellow color of the rhizomes and have many biological roles. The three primary curcuminoids in *C. longa* are curcumin, bisdemethoxycurcumin, and demethoxycurcumin (33). Curcumin has been extensively studied for its anti-inflammatory, antioxidant, and anticancer properties (34).

The essential oils in *C. longa* are responsible for its characteristic flavor and aroma (35). The primary components of these oils are turmerone, ar-turmerone, and curlone. Polysaccharides are the third major group of compounds in *C. longa* (36). The three main polysaccharides found in *C. longa* are curdlan, glycogen, and turmeric. These polysaccharides have demonstrated antiviral, anticancer, and immunomodulatory properties (37).

Dehydration is the primary method recommended for preserving turmeric quality throughout storage and usage, including freeze-drying, low-temperature drying, and microwave-vacuum drying techniques (38, 39). The hot air-drying method is a widely used and viable option due to its simplicity; however, prolonged exposure to high temperatures may degrade turmeric’s color, flavor, and bioactive compounds (40). While freeze-drying is considered the best preservation method for quality, it is expensive, time-consuming, and energy-intensive (38, 41). In comparison, sun-drying, which is regarded as a conventional technique, requires extended durations and often diminishes the product quality and bioactive components (42). Consequently, more efficient drying techniques are needed to maintain the desired quality and safeguard turmeric’s medicinal properties (42).

*C. longa* rhizomes comprise carbohydrates (69.4%), protein (6.3%), fats (5.1%), minerals (3.5%), and moisture (13.1%) (30). Essential oils extracted via steam distillation can reach approximately 5.8% of the rhizome content and contain compounds like borneol (0.5%), α-phellandrene (1%), zingiberene (25%), sabinene (0.6%), cineole (1%), and sesquiterpenes (53%) (30).

The primary bioactive compounds in *C. longa* are a blend of three curcuminoids: diferuloylmethane (94%, C_21_H_20_O_6_, curcumin I), demethoxycurcumin (6%, C_20_H_18_O_5_, curcumin II), and bis-demethoxycurcumin (0.3%, C_19_H_16_O_4_, curcumin III) (32). Curcumin I is considered a principal curcuminoid. The phenolic OH and CH_2_ groups in the β-diketone moiety of these compounds confer anti-inflammatory and antioxidant properties, among other bioactivities, making curcumin a valuable nutraceutical for chemo-preventive and therapeutic applications (23, 43).

Furthermore, curcumin is soluble in organic solvents such as methanol, ethanol, acetone, and dimethyl sulfoxide (DMSO) but is insoluble in water. Hence, solvent-based extraction is critical for optimal curcumin yield and environmental sustainability (44). Common solvents like methanol, ethanol, and acetonitrile are typically used to obtain antioxidative extracts from *C. longa*. Deep eutectic solvents have emerged as eco-friendly alternatives due to their non-toxic, biocompatible properties, minimal environmental impact, and enhanced efficiency in extracting bioactive compounds. These solvents, also known as designer solvents, can be synthesized for specific applications, including the extraction of bioactive chemicals (45).

Deep eutectic solvents are synthesized using various hydrogen bond donors and acceptors, such as choline chloride, menthol, and betaine (46). These compounds can be combined with sugars, carboxylic acids, alcohols, amines, or other hydrogen bond-containing compounds to form deep eutectic solvents. Key parameters influencing the synthesis of these solvents include solute-to-solvent ratio, extraction temperature, and duration (47).

Appropriate dosing significantly influences the biological efficacy of curcumin and turmeric extracts (48). Dietary administration of curcumin/turmeric extracts in suitable doses has been shown to inhibit tumor formation in multiple organs of mice and rats. Although high doses of curcumin and turmeric extracts have not increased mortality in mice, adverse effects have been observed in other species (49). For example, turmeric oleoresin administration in pigs has been reported to reduce feed conversion efficiency (or weight gain), increase liver and thyroid weights, and result in histological alterations in the kidney, liver, and urinary bladder. Similarly, high-dose turmeric extract administration in mice significantly changed tissue weights, weight gain, and red and white blood cell levels (49).

Curcumin and turmeric extracts are generally considered nontoxic and highly promising for various biological applications when administered at appropriate doses (50). Beyond the aforementioned biological activities, turmeric has been used in traditional medicine and contemporary and alternative medicine to manage conditions like anemia, indigestion, diabetes, hemorrhoids, edema, hepatitis, atherosclerosis, hysteria, wound healing, urinary diseases, psoriasis, rheumatism, anorexia, dermal diseases, inflammation, hepatic disorders, cough, and sinusitis (51, 52).

## 6 Techniques for curcumin isolation from turmeric rhizomes

Turmeric rhizomes contain two main pharmacologically active secondary metabolites: curcuminoids and essential oils (53). Demethoxycurcumin, bis-demethoxycurcumin, and curcumin are the primary compounds responsible for the biological activity of the rhizomes (54). Curcuminoids are extracted from turmeric rhizomes using traditional and modern techniques (43).

Traditional techniques include maceration and Soxhlet extraction (55, 56). Contemporary techniques include microwave extraction (57), enzyme-assisted extraction (58), pressurized fluid extraction (59), supercritical fluid extraction (60), and ultrasound extraction (61). Ethanol, dichloromethane, ethyl acetate, isopropanol, methanol, n-butanol, and acetone are the most commonly used solvents for curcuminoid extraction (54, 62, 63). Sahne et al. (55) used acetone for conventional and unconventional extraction because of its strong solubilization capability. Additionally, Muthukumar et al. (63) examined several organic solvents for curcumin extraction and identified acetone as the best solvent.

Curcumin can be extracted using thin-layer chromatography (TLC) on the extraction mixture, a traditional analytical method (54, 62). High-performance liquid chromatography (HPLC) is used to measure the curcumin content in the extract. After extraction, organic solvents are separated from the extract using a vacuum evaporator. The leftover material, or oleoresin, is then dissolved in methanol and analyzed using HPLC, as described by Yadav et al. (64).

The production and stability of curcumin are greatly influenced by the extraction method used. Several cutting-edge techniques for curcumin extraction from turmeric rhizomes have been investigated (55). Their results were compared with Soxhlet extraction, the most commonly used reference technique. The Soxhlet method was observed to achieve a significantly higher curcumin yield (6.9%) compared to enzyme-assisted (4.1%), ultrasound-assisted (3.92%), and microwave-assisted (3.72%) methods (55). The Soxhlet technique offers benefits such as low temperatures, faster extraction times, lower solvent quantities, and higher yields, which are unmatched by the newer extraction techniques (55). Although Soxhlet achieves higher yields (6.9%) than some modern methods like microwave-assisted extraction (3.72%) (55), its disadvantages outweigh this benefit. Advanced techniques offer a 90% reduction in processing time, 50–70% lower solvent consumption, and improved retention of bioactive properties (65).

Soxhlet extraction typically requires 4–6 h per cycle (66), with some processes extending beyond 24 h (67). This prolonged duration makes it impractical for industrial-scale applications where efficiency is critical. The method necessitates large volumes of organic solvents (e.g., ethanol, methanol) (65), raising both economic and environmental concerns due to solvent disposal requirements and potential ecological impacts. Also, continuous heating throughout the extraction cycle results in substantial energy consumption (67), making it cost-prohibitive compared to modern techniques. The prolonged exposure to elevated temperatures may degrade heat-sensitive curcuminoids (67), potentially compromising the bioactive integrity of the extract. On the other hand, the batch-processing nature of Soxhlet extraction hinders continuous production workflows, restricting its viability for commercial manufacturing (67).

Naksuriya et al. (68) investigated the kinetic degradation of curcumin from a naturally occurring curcuminoid mixture under different conditions with varying solvent dielectric constant, pH, and temperature. They also assessed the degradation of pure curcumin under similar settings, using a standard medium composed of a 50:50 (v/v) mixture of an aqueous buffer and methanol. The degradation kinetics of curcumin in the curcuminoid mixture showed a first-order response. The degradation rate increased simultaneously with the medium’s pH, temperature, and dielectric constant (65).

Curcumin underwent rapid degradation by autoxidation in an aqueous buffer (pH: 8) at a steady rate of 0.28−1 h, resulting in a half-life (t1/2) of 2.5 h (65). Mixing curcumin with ω-methoxy poly (ethylene glycol)-b-(N-(2-benzoyloxypropyl) methacrylamide) polymer micelles improved its stability, increasing it approximately 300–500 times compared to pure curcumin in a phosphate buffer and methanol mixture (65). Thus, this stabilization approach offers the potential for developing formulations suitable for further pharmacological and clinical studies (68).

Another study by Liu et al. (69) investigated the use of naturally occurring organic acids and sugars to produce deep eutectic solvents for curcuminoid extraction. Under ideal conditions (temperature: 50°C, solid-to-liquid ratio: 0.1/10 g/mL, and extraction time: 30 min), a solvent containing a 1:1 ratio of citric acid and glucose with 15% water yielded higher extraction efficiency compared to conventional solvents. This process is a promising substitute for extracting natural coloring agents because it is eco-friendly and sustainable (69).

When purifying and separating curcuminoids from the oleoresin, volatile turmeric oil (CP-01) dissolves curcumin, causing issues with recrystallization. To address this, mixtures of several organic solvents were evaluated for the selective recrystallization of curcuminoids (54). A combination of isopropyl alcohol and hexane in a volumetric ratio of 1:1.5 was identified as the optimal solvent for recrystallizing curcuminoids, yielding a recrystallized powder with a purity of up to 99.45% w/w (54). In comparison, the raw curcuminoid powder had a curcumin content of 76.82% w/w (54).

In a study by Ahmed et al. (70), the authors focused on quantifying curcumin from *C. longa* roots and commercial powder using a green chromatography approach. This method, emphasizing environmental sustainability by minimizing harmful solvents, aligns with renewed interest in green analytical techniques. The proposed method underwent validation according to ICH guidelines, assessing system suitability, linearity, precision, and accuracy. Its simplicity is further enhanced by short retention times, the use of an eco-friendly mobile phase (ethanol: water), and a UV-Vis detector. Overall, this method is more environmentally friendly than previously reported techniques, making it suitable for routine, eco-conscious analysis of curcumin.

## 7 Physicochemical properties of curcumin

According to Nelson et al. (71), turmeric contains up to ~5% curcumin (1,7-bis-(4-hydroxy-3-methoxyphenyl)-1,6-heptadiene-3,5-dione) (72), also known as diferuloylmethane (73). Curcumin is lipophilic with a strong affinity for fats and oils. It is water-insoluble and also insoluble in acidic or neutral solutions (70). However, it is soluble in organic solvents, like ethanol, dimethylsulfoxide, and acetone, which can be used to extract it from turmeric rhizomes. Its molecular weight is 368.38 g/mol, and its chemical formula is C_21_H_20_O_6_ (70). Structurally, it comprises three primary functional groups: (i) two aromatic ring systems with an alpha, beta-unsaturated beta-diketone moiety, (ii) one o-methoxy phenolic group, and (iii) a seven-carbon linker (74).

Curcumin has a melting point of 183°C and exhibits diketone/keto-enol tautomerism due to its β-diketone moiety (75, 76). The balance between diketone and keto-enol forms is strongly influenced by temperature, pH, and solvent polarity (77), and the enol-to-keto ratio is a significant determinant of curcumin’s pharmacological properties (78). Under acidic and neutral pH conditions, curcumin adopts the keto form, exhibiting chemical stability and acting as a proton donor (79). At pH more than 7, it is unstable and shifts to the enol form, an electron donor that contributes to its antioxidant effects (79, 80).

The lipophilic nature of curcumin is collectively attributed to its nonpolar methyl groups, aromatic rings, and aliphatic bridge (81). However, its three hydroxyl groups undergo protonation and deprotonation depending on pH, which affects its water solubility (81). In neutral and acidic environments, curcumin has poor water solubility due to hydroxyl group protonation. Under alkaline conditions, deprotonation leads to negative charges, increasing water solubility (82). These changes are reflected in shifts in curcumin’s log *P* value, which decreases (from 3.2) with increased polarity upon hydroxyl group deprotonation, enhancing its water solubility and facilitating its elimination (83).

Manolova et al. (84) utilized advanced ultraviolet-visible spectroscopy (UV-VIS) and quantum chemical calculations to investigate curcumin’s tautomerism in ethanol/water binary solutions (84). Their findings indicate that curcumin in ethanol exists only in the enol-keto tautomer, while water induces a shift to the diketone tautomer (84). Mass spectrometry and liquid chromatography studies confirmed that the enol form predominates in water/acetonitrile solutions (85). In nonpolar solvents such as carbon tetrachloride, curcumin remains in its enol tautomer in solid and liquid states. In solution, curcumin is inherently unstable, with a vibrant yellow color that transitions to deep crimson when exposed to alkaline conditions (86).

Structure-activity relationship studies have highlighted that the two phenyl rings connected by a C-7 linker with keto-enol functionality are crucial for curcumin’s biological activity (87). The unsaturation in the linker, which provides conformational flexibility, is particularly important for its antitumor and anticancer effects, though it is less critical for its redox regulatory or apoptotic activities (80). While synthetic methods for producing curcumin from acetylacetone and vanillin have been established, these approaches typically require lengthy reaction times and yield low amounts of product over multiple steps (88). As a result, there is a need for more efficient and streamlined synthetic methods to produce curcumin quickly and effectively (89).

## 8 Bioavailability of curcumin

The primary limitation in utilizing curcumin’s therapeutic potential stems from its intrinsic physicochemical characteristics. These characteristics restrict its functional efficacy, rendering less than 2% of curcumin bioavailable to the body and limiting its clinical use (71, 90–92).

### 8.1 Intestinal stability and permeability of curcumin

**Figure 3:**
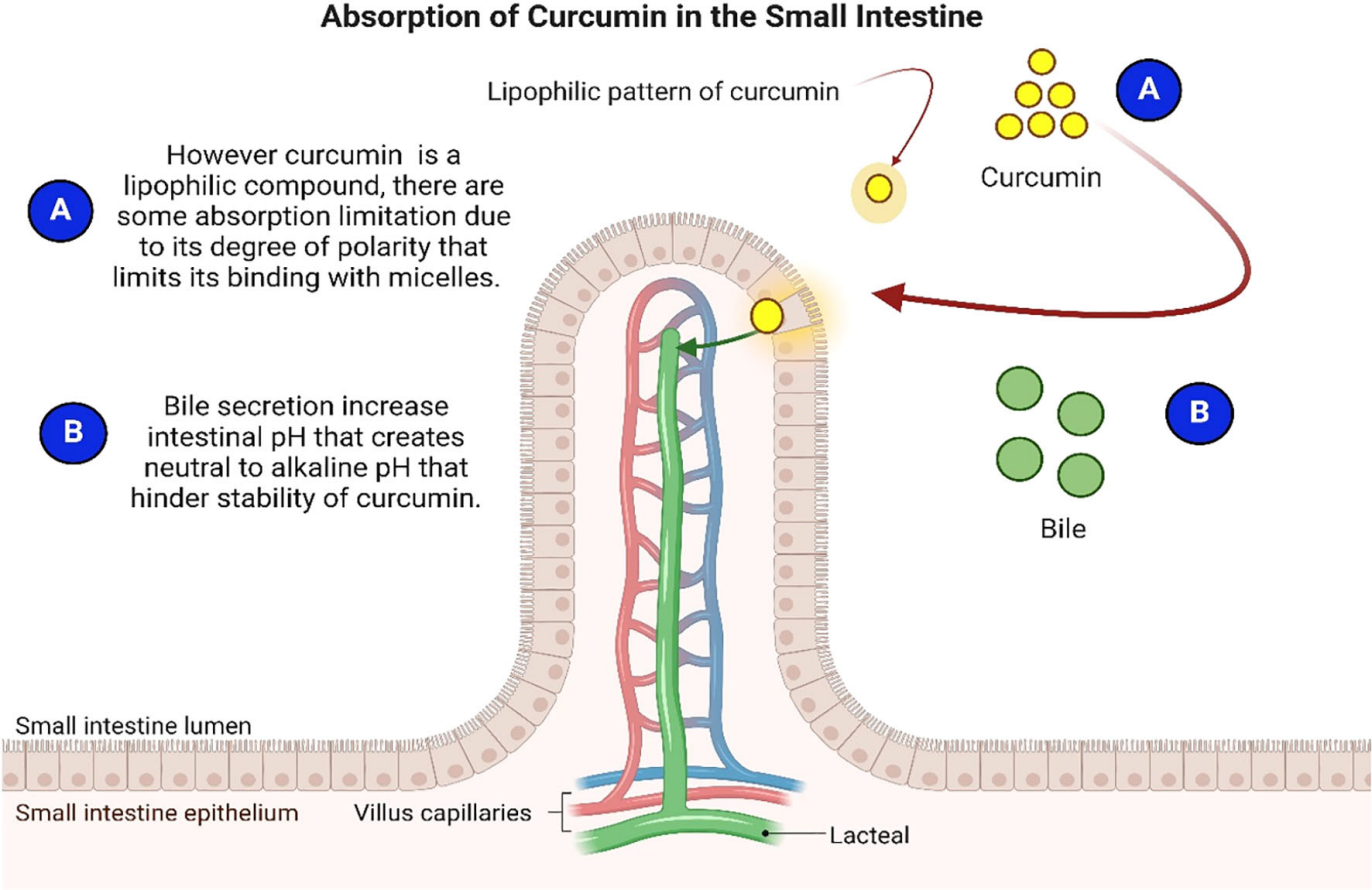
Absorption of curcumin in small intestine.

Curcumin’s stability in the intestine is critical to its permeability and absorption. Several physicochemical properties influence its intestinal stability, posing significant challenges for absorption and thus reducing its therapeutic potential across body tissues (86). These challenges include curcumin’s poor solubility in gastrointestinal fluids, which hinders its passage through the mucus layer and subsequent absorption by epithelial cells. Like other lipophilic compounds, only stable and soluble curcumin components post-digestion are absorbed by enterocytes (93). The metabolism of lipophilic nutrients follows a different absorption pathway through the membrane compared to polar nutrients, majorly due to the non-polarity of lipophilic compounds (94). **Figure 3** illustrates the process of curcumin absorption in the small intestine.

Lipophilic compounds are transported through membranes by encapsulation with micelles, which the body produces naturally (94). These micelles have polar exteriors and nonpolar interiors, allowing them to bind to nonpolar compounds and facilitate membrane transport (94). As a lipophilic compound, curcumin is expected to follow this pathway; however, its limited polarity can hinder bindings with micelles (94). Upon entering the small intestine, bile secretion increases the pH to a neutral or alkaline state, which reduces curcumin’s stability and solubility, further interfering with its absorption (95).

The intestinal barrier plays a key role in curcumin’s entry into the bloodstream. Although limited information exists on the exact mechanisms, recent studies suggest passive diffusion as the primary mechanism for cellular uptake of natural curcumin, including clathrin-mediated endocytosis (96–98). Notably, curcumin uptake was observed to have a concentration-dependent effect. At relatively lower concentrations, passive transport dominates, while higher concentrations trigger active transport mechanisms, leading to reduced absorption rates into the ileum (99).

At low concentrations, curcumin primarily interacts with the polar heads of the outer membrane surface. Conversely, at higher concentrations, it tends to accumulate within the nonpolar chains of the phospholipid bilayer (100, 101). This accumulation may result in reduced membrane fluidity, as observed in several studies (101, 102). *In vivo* studies on rats showed poor absorption of orally administered curcumin, with more than 90% excreted in feces within 72 h (92).

Plasma concentrations remained minimal, even at high doses, with most curcumin localized in the small intestine and little reaching systemic circulation, resulting in a bioavailability of approximately 1% (71, 90, 92). Human studies corroborate these findings, with low plasma concentrations of curcumin and its metabolites even at high doses, with bioavailability ranging from 0.16 to 1% (92). Poor curcumin permeability was attributed to intestinal first-pass metabolism and intracellular retention (103). While rodent studies provide valuable findings, their extrapolation to human oral consumption remains unclear, necessitating further research and evidence for validation.

### 8.2 Metabolism and elimination of curcumin

**Figure 4:**
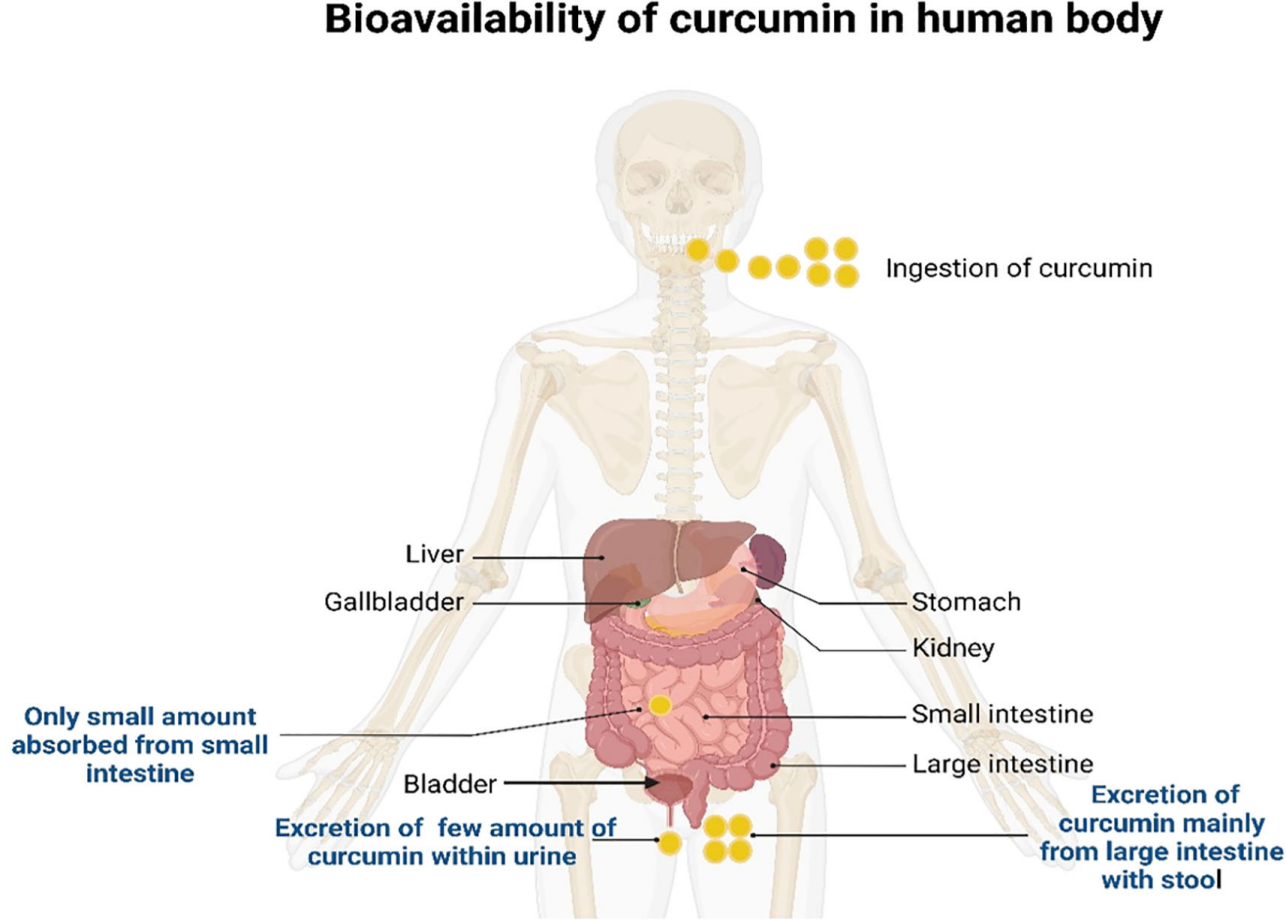
Bioavailability, absorption, and excretion of curcumin in the human body.

Curcumin’s bioavailability in humans is mostly limited by poor intestinal absorption, rapid hepatic metabolism, and rapid systemic elimination, even at high doses of 12 g/day (104). Most orally ingested curcumin is excreted in feces without undergoing significant metabolic transformation (98). However, the small fraction that is absorbed undergoes a two-stage metabolic pathway. During the initial phase, reductase levels are decreased in enterocytes and hepatocytes (98). Curcumin elimination mostly occurs via feces rather than urine, and approximately 90–98% of orally administered curcumin is eliminated through feces and bile (92, 105, 106). The bioavailability, absorption, and excretion of curcumin inside the human body are indicated in **Figure 4**.

The remaining curcumin is absorbed by enterocytes, where most of it undergoes xenobiotic metabolism via intestinal and hepatic cell-resident phase I and II enzymes (107, 108). During phase I metabolism, dihydrocurcumin (2HC) is produced when reductases break the double bonds in curcumin, followed by tetrahydrocurcumin (4HC), hexahydrocurcumin (6HC), and octahydrocurcumin (8HC) (108). Phase I metabolism also involves cytochrome P450 (CYP) enzymes, particularly CYP3A4, which is the most prevalent hepatic enzyme and plays a key role in metabolizing dietary phytochemicals like curcumin (109–111).

Dei Cas and Ghidoni (74) reported that 2HC, 4HC, 6HC, and 8HC are produced after reduction. The enzymes involved are nicotinamide adenine dinucleotide phosphate (NADPH)-dependent reductase, alcohol dehydrogenase, and an unknown microsomal enzyme that sped up curcumin degradation (112). Hassaninasab et al. (113) analyzed an enzyme isolated from *Escherichia coli* that reduces curcumin. They found two reduction phases in the microbial degradation of curcumin by the purified enzyme. Curcumin was initially changed into intermediate 2HC and, ultimately, 4HC, depending on NADPH availability (113).

Curcumin and its reduced metabolites undergo conversion to glutaric acid and sulfate in both *in vitro* and *in vivo* conditions. These transformations are facilitated by glucuronyl transferase and sulfotransferase (SULT) enzymes, which mediate glucuronidation and sulfation processes. Sulfated and glucuronidated curcumin has been identified in the intestines and livers of both rats and humans (74), while human plasma contains water-soluble conjugates of sulfate and glucuronide, indicative of curcumin absorption following oral administration (70).

Hager et al. (114) reported that curcumin glucuronidation is catalyzed by uridine diphosphate-glucuronosyltransferase (UDP-UGT). Meanwhile, human phenol SULT1A1 and SULT1A3 are metabolized in rat intestines. Notably, these metabolic modifications result in reduced biological activity (108). Reduced or conjugated curcumin forms, such as 4HC, 6HC, and curcumin sulfate, showed a diminished ability to inhibit cyclooxygenase-2 (COX-2) synthesis (108). Additionally, while other conjugates exhibited decreased inhibition of prostaglandin E2 synthesis, hexahydrocurcuminol is biologically inert (115).

Compared to curcumin itself, the biological effectiveness of curcumin metabolites, excluding 4HC, is considerably reduced (82, 116). Many strategies have been developed to enhance curcumin absorption, including the use of piperine, which inhibits glucuronidation, and the incorporation of curcumin into delivery systems such as liposomes, nanoparticles, phospholipid complexes, or structural analogs of curcumin (110, 111).

Pfeiffer et al. (117) demonstrated the formation of reductive metabolites, such as 4HC, 6HC, and 8HC, during curcumin metabolism in rat liver tissue sections (117). In phase II metabolism, which occurs in the intestinal and hepatic cytosol, UDP-UGT and SULT enzymes catalyze the conjugation of glucuronide and sulfate to curcumin and its reduced metabolites (107, 108). Specifically, curcumin undergoes sulfation mediated by SULT1A1 and SULT1A3 and glucuronidation facilitated by UDP-UGT enzymes (107, 108, 118). This yields curcumin glucuronide (CG), HC glucuronide (2HC-G), 4HC glucuronide (4HC-G), 6HC glucuronide (6HC-G), and 8HC glucuronide (8HC-G), with corresponding sulfate conjugates produced similarly. Notably, glucuronide conjugates are approximately twice as abundant as sulfate conjugates (119).

The principal human curcumin metabolites following oral consumption are predominantly 4HC, 6HC, and glucuronide conjugates such as CG, 4HC-G, and 6HC-G (107, 108, 115). Pan et al. (118) conducted hydrolysis of plasma curcuminoid samples using glucuronidase, revealing that 99% of curcumin metabolites in plasma are glucuronide conjugates (118). These metabolic modifications significantly alter curcumin’s physicochemical properties, shifting its polarity and consequently increasing its water solubility. This shift is evident through curcumin’s log *P* value, which decreases from 3.2 to 1.6 when conjugated with glucuronide, enhancing its water solubility and facilitating urinary elimination (83). Similar changes are observed with curcumin’s reduced metabolites. For instance, the log *P* value of 6HC decreases from 2.2 to 1.1 upon glucuronidation (83). Thus, phase I and II metabolism reduces curcumin’s bioavailability and alters the structural and chemical interactions of curcumin and its metabolites (119).

Transporters significantly influence curcumin’s bioavailability by regulating its efflux and influx across cellular barriers (114). Key efflux transporters involved in the transport of curcumin and its metabolites include P-glycoprotein (P-gp), multidrug resistance-associated proteins (MRP), and breast cancer resistance protein (BCRP) (114). These transporters are widely distributed in tissues such as the epithelial cells of the gastrointestinal tract, the blood-brain barrier, and the liver (120). After absorption into enterocytes, soluble curcumin and its metabolites face two potential pathways: efflux back into the intestinal lumen via transporters, such as MRP2, BCRP, and P-gp, or movement into the portal blood through MRP1 and MRP3 transporters (92, 121). Curcumin and its metabolites undergo further metabolism in the liver before being excreted into bile or distributed to tissues or kidneys via systemic circulation (92, 121).

Efflux transporters play a crucial role in limiting intracellular drug accumulation, thereby reducing drug efficacy (120). The dual role of these transporters in facilitating curcumin’s cellular movement underscores their significance when developing strategies to enhance curcumin’s bioavailability (114).

### 8.3 Tissue distribution and half-life of curcumin

The half-life of curcumin is a key parameter for understanding its pharmacokinetics. In diabetic rats, the half-life of curcumin in systemic circulation following oral intake was 32.70 ± 12.92 min (122). This finding aligns with a prior study on healthy rats, which reported an elimination half-life of 28 min after oral ingestion of curcumin (91). These short half-life values emphasize curcumin’s rapid metabolism and elimination, contributing to its limited systemic bioavailability and therapeutic efficacy after oral intake (117). Maintaining therapeutic levels of curcumin in systemic circulation remains a significant challenge due to this rapid clearance. In humans, calculating the absorption rate and elimination half-life for orally administered curcumin remains challenging, as serum levels often fall below the detection limit (123). However, understanding curcumin’s tissue distribution is essential for optimizing its therapeutic potential, especially in target body regions (117).

A recent review of curcumin’s distribution reported that although curcumin’s tissue distribution has been extensively studied in rats, evaluations in humans are limited (71). Studies using rodent models have shown variable tissue distribution patterns attributed to differences in dose preparations, extraction methods, and detection assays (67). This variability is compounded by curcumin’s rapid degradation and transformation both before and after absorption, complicating the consistency of results and observed distribution patterns (71). Ultimately, these findings suggest that the parent compound does not accumulate significantly in specific organs (71).

While numerous clinical studies in humans have assessed curcumin’s systemic effects following oral intake, none have evaluated its tissue distribution. In rodent studies, orally administered curcumin was primarily detected in the stomach and small intestine, accounting for approximately 90% of the curcumin, with only trace amounts of unchanged curcumin in the liver and kidney (124, 125). At 24 h, only 1% of curcumin remained in the stomach and small intestine (124, 125). Some studies have used radioactive-labeled curcumin in rats to address the difficulties in measuring accurate tissue distribution from orally administered curcumin. These investigations revealed detectable radioactivity in the blood, liver, and kidney after curcumin administration, with consistent absorption percentages regardless of the administered dose (126).

Similarly, in a separate study, mice injected intraperitoneally with radioactive carbon (^14^C) curcumin showed peak radioactivity levels in various tissues, with the liver and intestinal mucosa having the highest peaks. Subsequently, radioactivity rapidly declined to 20–33% of the peak values within 4 h for most tissues, except the small intestine, which showed a slower decline within 8 h (127). These findings highlight the limitations of using rat models to infer human tissue distribution due to interspecies differences in drug metabolism and pharmacokinetics (128). Consequently, further studies are needed to accurately examine tissue distribution in humans following oral curcumin intake.

### 8.4 Bypassing curcumin’s poor bioavailability

The physicochemical properties of curcumin significantly limit its clinical utility, as its poor bioavailability restricts its functional capabilities (123). Thus, understanding the challenges associated with curcuminoids’ bioavailability is key to developing effective strategies to overcome these limitations (124). One primary factor contributing to curcumin’s low bioavailability is its rapid conjugation, particularly glucuronidation in the intestine and liver, facilitated by UDP-UGT and accounting for more than 80% of curcumin’s metabolism (129).

Enhancing curcumin’s therapeutic potential requires a focus on prolonging its serum bioavailability by increasing its half-life and reducing its metabolic rate (124, 125). Improving curcumin’s bioavailability would require strategies that inhibit metabolic pathways and slow curcumin’s elimination rate. The coadministration of bio-enhancing substances that modulate these pathways has been widely investigated (125). Piperine stands out for its significant potential to improve the systemic availability of curcumin and, hence, its bioavailability (130). Consequently, formulations integrating curcumin with piperine have gained attention as a viable approach to overcoming curcumin’s poor bioavailability (125).

**Figure 5:**
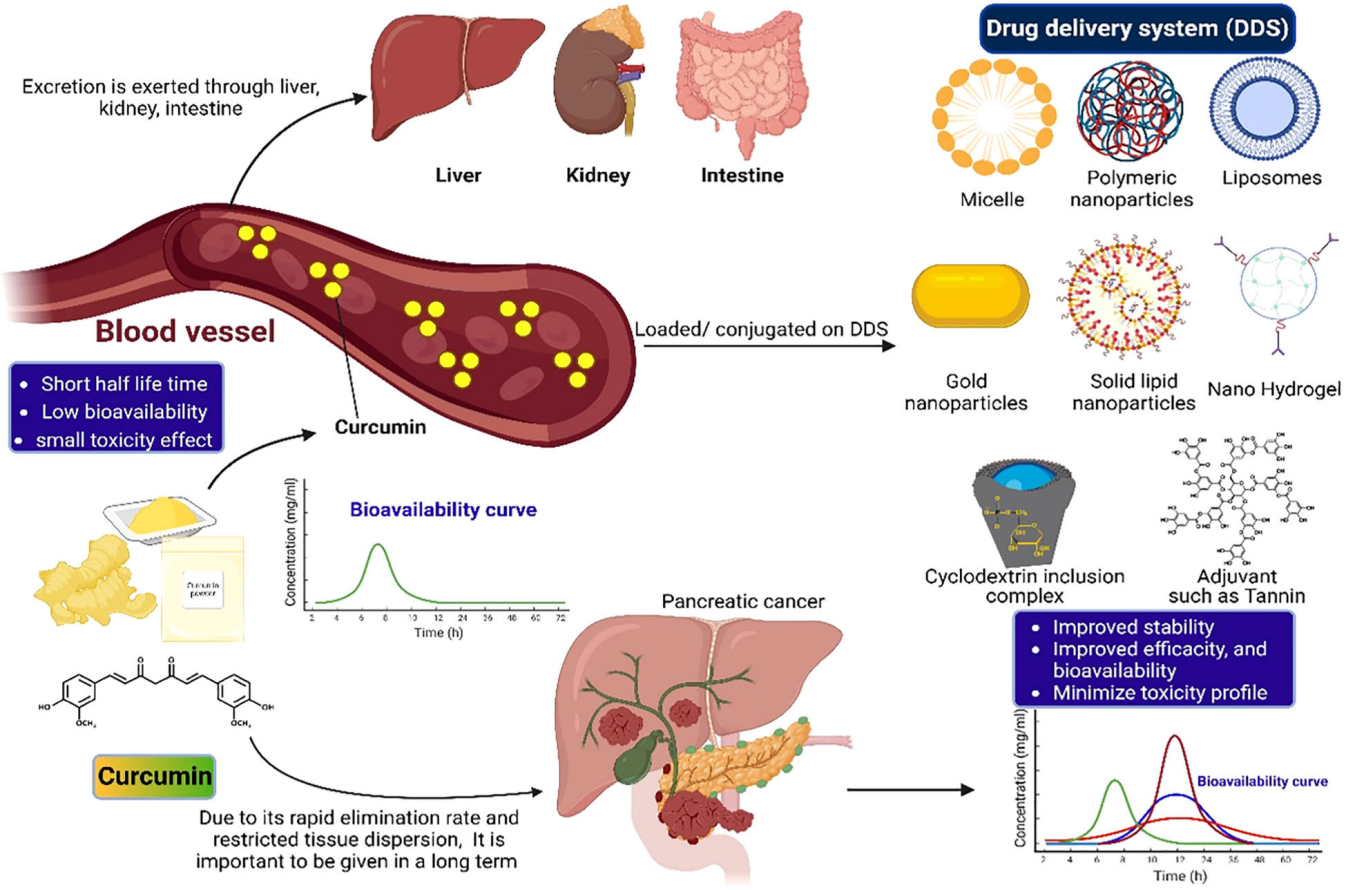
Improvement of curcumin bioavailability through incorporation into medication delivery systems.

**Figure 5** illustrates the improvement of curcumin bioavailability via integration with drug delivery systems.

### 8.5 Current strategies to enhance bioavailability

While curcumin demonstrates immense therapeutic promise, its clinical application is significantly hampered by poor bioavailability due to limited absorption, rapid metabolism, and quick systemic elimination. Researchers and clinicians are actively exploring various strategies to overcome these challenges, with innovative formulation techniques and delivery systems offering practical insights. These strategies encompass the procedures outlined below:

#### 8.5.1 Adjuvants and combinations

One of the simplest strategies involves co-administration with adjuvants that inhibit curcumin’s metabolism or enhance its absorption (131). The most well-known example is piperine, a compound found in black pepper. Piperine is known to inhibit enzymes involved in curcumin’s metabolism, thereby increasing its systemic availability. Clinical trials have shown that curcumin combined with piperine can significantly increase curcumin levels in the blood (132).

#### 8.5.2 Nanotechnology-based delivery systems

Nanotechnology offers a revolutionary approach to improve curcumin’s solubility, stability, and bioavailability by encapsulating it within nanocarriers (133). These systems can bypass gastrointestinal barriers and enhance the compound’s reach to target sites (134).

##### 8.5.2.1 Liposomes

These artificial vesicles encapsulate curcumin, making it easier for the body to absorb. Liposomal formulations have demonstrated higher bioavailability compared to free curcumin. They are composed of phospholipid bilayers that can encapsulate curcumin within aqueous compartments or lipid bilayers, enabling targeted delivery and minimizing off-target effects (135).

##### 8.5.2.2 Polymeric nanoparticles

These customizable platforms, often made from biocompatible polymers like poly (lactic-co-glycolic acid) (PLGA) and polyethylene glycol (PEG) derivatives, can encapsulate curcumin to improve its solubility and stability (136). They allow for controlled release kinetics, enhanced cellular uptake, and prolonged circulation times, optimizing therapeutic efficacy.

##### 8.5.2.3 Solid lipid nanoparticles

Solid lipid nanoparticles are biocompatible lipid-based nanocarriers that offer stability and sustained release properties, making them suitable for encapsulating hydrophobic compounds like curcumin (137).

##### 8.5.2.4 Polymeric micelles

These self-assembling colloidal systems spontaneously form when surfactants are dispersed in water. Curcumin can be solubilized within its hydrophobic core, significantly enhancing its bioaccessibility and potentially increasing the permeability of epithelial cells. Micellar curcumin formulations have shown dramatically higher bioavailability in human studies (138, 139).

##### 8.5.2.5 Nanoemulsions

These oil-in-water or water-in-oil formulations provide a stable platform for delivering hydrophobic compounds like curcumin, enhancing its bioavailability and therapeutic efficacy.

##### 8.5.2.6 Cyclodextrin inclusion complexes

Cyclodextrins are cyclic oligosaccharides that can form inclusion complexes with curcumin, improving its solubility and stability (140). This technique has shown improved bioavailability and enhanced antiproliferative effects (141).

##### 8.5.2.7 Nanogels

These crosslinked polymer networks can enhance curcumin’s solubility, improve its controlled release, and prolong its half-life, leading to increased bioavailability and improved therapeutic effects (142).

#### 8.5.3 Specific curcumin formulation

Various specialized formulations have been developed and tested in clinical trials to enhance bioavailability, BCM-95^®^CG Biocurcumax™, in formulation, combines curcuminoids with essential oils from turmeric rhizome, demonstrating significantly improved bioavailability compared to standard curcumin (143). Furthermore, CuraMed^®^ and Curamin^®^ products often incorporate BCM-95^®^ and other synergistic ingredients to enhance absorption and therapeutic effects (144). Also, Curcuwin^®^ and CurQfen^®^ formulations utilize hydrophilic carriers or fenugreek dietary fibers to improve curcumin’s systemic availability (145).

#### 8.5.4 Novel strategies to enhance bioavailability

Beyond current approaches, cutting-edge research is exploring even more innovative methods.

##### 8.5.4.1 SMEDDS

These systems are isotropic mixtures of oils, surfactants, co-surfactants, and drugs that form fine oil-in-water emulsions or microemulsions upon gentle agitation in aqueous media. This pre-dispersion in the gastrointestinal tract can significantly enhance the dissolution and absorption of poorly soluble drugs like curcumin by presenting them in a finely dispersed, absorbable form (146).

##### 8.5.4.2 Prodrug approaches

Designing curcumin prodrugs involves chemically modifying the curcumin molecule to improve its physicochemical properties, such as water solubility and membrane permeability. These prodrugs are designed to be inert until metabolized *in vivo* to release the active curcumin, potentially at the target site, thereby overcoming bioavailability issues (147).

##### 8.5.4.3 Co-crystallization

This technique involves forming crystalline solids composed of curcumin and a co-former molecule in a defined stoichiometric ratio. Co-crystals can improve the solubility and dissolution rate of curcumin without altering its chemical structure, leading to enhanced bioavailability (148).

##### 8.5.4.4 Amorphous solid dispersions

By dispersing curcumin in an amorphous state within a polymer matrix, amorphous solid dispersions prevent crystallization and maintain curcumin in a higher energy, more soluble form. This amorphous state can significantly enhance the dissolution rate and saturation solubility, leading to improved absorption (149, 150).

##### 8.5.4.5 Targeted delivery systems with ligands

Incorporating specific ligands (e.g., antibodies, peptides, folate) onto the surface of nanocarriers can enable active targeting of curcumin to specific cells or tissues that overexpress certain receptors. This not only enhances accumulation at the disease site but also potentially improves cellular uptake and efficacy while minimizing off-target effects and systemic exposure to high doses (151).

##### 8.5.4.6 Microencapsulation and spherical crystallization

These techniques aim to encapsulate curcumin within micro-sized particles or create spherical agglomerates to improve flowability, compressibility, and dissolution rate, indirectly contributing to better absorption (152).

#### 8.5.5 General limitations of bioavailability enhancement strategies for curcumin

While significant progress has been made in enhancing curcumin’s bioavailability, several limitations and challenges are associated with the current and novel strategies (153).

##### 8.5.5.1 Complexity and cost of manufacturing

Many advanced formulations, particularly those involving nanotechnology (liposomes, polymeric nanoparticles, SMEDDS), require complex manufacturing processes, specialized equipment, and stringent quality control. This can lead to higher production costs, potentially making the final product less accessible or affordable for widespread use (153).

##### 8.5.5.2 Scalability issues

Translating laboratory-scale production of nanoformulations to industrial-scale manufacturing can be challenging. Ensuring consistent particle size, stability, and drug loading at larger scales requires significant investment and expertise (153).

##### 8.5.5.3 Stability concerns

While some formulations improve stability, others might introduce new stability issues. For example, liquid SMEDDS can suffer from stability and leakage problems, though solid SMEDDS aims to address this. Amorphous solid dispersions, while improving solubility, have a natural tendency to transform into a more stable crystalline form over time, which can lead to a loss of the bioavailability benefit (153).

##### 8.5.5.4 Toxicity of excipients and carriers

Some excipients or carrier materials used in these formulations, especially surfactants in SMEDDS or certain polymers in nanoparticles, might have inherent toxicity concerns, particularly with long-term use or at high doses. Immunotoxicity assessment is crucial for nanoparticles, as their intrinsic properties can influence potential adverse effects on the immune system (153).

##### 8.5.5.5 Regulatory hurdles

Novel drug delivery systems and formulations often face rigorous regulatory scrutiny. Demonstrating long-term safety, efficacy, and batch-to-batch consistency for these complex systems can be a lengthy and expensive process (153).

##### 8.5.5.6 Predictive *in vitro* models

A lack of robust and predictive *in vitro* models to assess the performance of these complex formulations remains a significant limitation. This makes it difficult to accurately predict *in vivo* behavior from laboratory data, necessitating more extensive animal and human trials (153).

**Table 1** presents the advanced strategies that may be utilized to improve the bioavailability of curcumin.

**Table 1:** Advanced strategies to enhance curcumin bioavailability.

Strategy	Formulation type	Key components/techniques	Enhancement of bioavailability	References
Lipid-based systems	Nano emulsions	MCT oil, tween 80, lecithin	40-50×	(154)
Lipid-based systems	Solid lipid nanoparticles (SLNs)	Glyceryl monostearate, poloxamer 188	25-30×	(137)
Lipid-based systems	Self-microemulsifying drug delivery systems (SMEDDS)	Capryol 90, labrasol, transcutol HP	55-60×	(146)
Polymeric nanoparticles	Poly (lactic-co-glycolic acid) nanoparticles	PLGA-PEG, PVA stabilizer	15-20×	(155, 156)
Polymeric nanoparticles	Chitosan nanoparticles	Chitosan, TPP crosslinker	10-12×	(157, 158)
Cyclodextrin complexes	HPβCD inclusion complex	Hydroxypropyl-β-cyclodextrin	8-10×	(159, 160)
Phospholipid complexes	Phytosomes (Meriva®)	Soy phosphatidylcholine	20-25×	(161, 162)
Micellar systems	Polymeric micelles	Pluronic F127, Soluplus	30-35×	(138, 163)
Nanosuspensions	Wet milling	HPMC, poloxamer 407	12-15×	(164, 165)
Liposomes	PEGylated liposomes	DSPE-PEG, cholesterol	20-25×	(166, 167)
Prodrugs	Curcumin-glucuronide conjugate	Glucuronidation to bypass metabolism	5-8×	(147)
Hybrid nanoparticles	Gold-curcumin nanoparticles	AuNPs, citrate coating	10-12×	(168, 169)
Exosome encapsulation	Milk exosomes	Bovine milk-derived exosomes	50-60× (BBB targeting)	(170)
Biopolymer hydrogels	Alginate-chitosan beads	Alginate, Ca²^+^ crosslinking	6-8×	(171)
Co-administration	Piperine synergy	Black pepper extract (CYP3A4/P-gp inhibition)	20×	(172, 173)
Structural analogues	EF24 (curcumin analog)	Difluorinated curcumin (higher stability)	30× (anticancer potency)	(174, 175)
Cocrystals	Curcumin-vanillin cocrystal	Hydrogen-bonded co-formers	5-7×	(176)
Electrospun fibers	PVA-curcumin nanofibers	Polyvinyl alcohol (oral fast-dissolving films)	8-10×	(177, 178)
Pickering emulsions	Silica-stabilized emulsion	Mesoporous silica nanoparticles	15-18×	(179)
Mucoadhesive systems	Carbopol-based gel	Carbopol 934P, Noveon AA-1	10× (localized delivery)	(180)
Inhalable nanoparticles	PLGA dry powder inhaler	Mannitol as carrier	25× (lung targeting)	(181)
Floating microspheres	Alginate microballoons	Sodium alginate, CaCO_3_ (gastric retention)	12×	(182, 183)
CRISPR-Cas9 delivery	Curcumin-gRNA complexes	Lipid nanoparticles for gene regulation	10×	(184, 185)

MCT, Medium-chain triglyceride oil; PLGA, poly(lactic-co-glycolic acid); PEG, polyethylene glycol; PVA, polyvinyl alcohol; TPP, sodium tripolyphosphate; SLNs, solid lipid nanoparticles; SMEDDS, Selfmicroemulsifying drug delivery systems; EF24, 3,5-bis(2-fluorobenzylidene) piperidin-4-one; CYP3A4, cytochrome P450 3A4; P-gp, P-glycoprotein; DSPE-PEG , 1,2-Distearoyl-sn-glycero-3-phosphoethanolamine-Polyethylene glycol; HPMC, hydroxypropyl methylcellulose.

## 9 Chemical degradation of curcumin

Curcumin’s extensive health benefits, minimal side effects, and low supply cost have driven significant research efforts to develop it as a supplement, therapeutic food product, or potentially a pharmaceutical product (186). Its popularity has contributed to a growing global trend of commercial food and non-food products containing turmeric, including beverages, supplements, creams, and soaps (186).

However, curcumin is highly susceptible to chemical degradation, which limits its stability and bioavailability (126). This is particularly problematic for oral ingestion because the gastrointestinal tract is unfavorable for curcumin’s stability, as will be discussed below.

### 9.1 Alkaline degradation of curcumin

The structural stability of curcumin may diminish under alkaline conditions. In basic environments, it undergoes hydrolytic degradation and alpha, beta-unsaturated beta-diketone moiety cleavage (88). This degradation gives rise to trans-6-(4’-hydroxy-3’-methoxyphenyl)-2, 4-dioxo-5-hexanal, which then undergoes further cleavage to yield compounds like ferulic acid, feruloymethane, and vanillin (95).

Reports have shown that 90% of curcumin degradation under alkaline and neutral conditions occurred within 15–30 min, while in acidic incubations, degradation was substantially slower, with less than 20% of total curcumin degraded within 60 min (71, 92). This suggests the importance of pH when considering the development of curcumin-based functional food products (85).

### 9.2 Autooxidation of curcumin

Curcumin is also susceptible to autooxidation, which occurs through radical chain reactions and spontaneously occurs in aqueous solutions (92). Autoxidation occurs at physiological pH and is initiated by the surrounding free radicals, which autoxidize the phenolic hydroxyls on the curcumin molecule (74). Autooxidation produces a succession of bicylopentadione compounds, where the seven-carbon chain is oxygenated and doubly cyclized (74), hence yielding bicyclopentadione, vinyl ether, and spiro epoxide (74, 92). Additionally, during curcumin autoxidation, small amounts of two configurational isomers of bicyclopentadione are produced as by-products of lipoxygenase-catalyzed oxygenation (187).

### 9.3 Photodegradation of curcumin

Curcumin is susceptible to photodegradation when exposed to light in both crystalline and solubilized forms (128). This is visually detected as color fading. Chemical degradation of curcumin occurs at the alpha, beta-unsaturated beta-diketone moiety, yielding compounds such as vanillin, vanillic acid, p-hydroxybenzaldehyde, ferulic aldehyde, and ferulic acid (188).

## 10 Characterization of curcumin

Curcuminoids are extensively employed in the food processing and pharmaceutical sectors due to their unique properties. Hence, accurate identification and characterization of curcuminoids in various substances are crucial, with the selection of an appropriate analytical technique influenced by factors such as sample type, analytical objectives, and detection limits (189, 190). Chromatography and electrophoresis-based methods are among the preferred approaches for curcuminoid analysis (130).

According to Nurjanah and Saepudin (62) TLC is one method used to fractionate turmeric extracts. Although it is selective, easy to use, and cost-effective, its limitations—such as low resolution and long separation times—have reduced its popularity in turmeric research (62). Recent advancements in TLC have introduced new high-performance TLC (HPTLC), which effectively addresses the constraints associated with traditional TLC (191). HPTLC provides many benefits, including enhanced resolution, reduced detection limit, and increased image scanning capabilities (190).

HPLC remains the most widely used chromatographic technique for qualitative and quantitative analysis of curcumin. Curcuminoids may be analyzed using different HPLC techniques. When combined with a UV–VIS detector, HPLC offers high precision, accuracy, and sensitivity (129). For more complex matrices, liquid chromatography-mass spectrometry techniques have been developed to detect and measure curcumin traces in food, biological fluids, and other samples (189). Several liquid chromatography-mass spectrometry (LC/MS) techniques have been established to identify and measure curcumin in various matrices (192, 193).

Tandem mass spectrometry and ultra-HPLC have demonstrated high throughput, sensitivity, and selectivity for curcuminoid quantification, significantly reducing analytical time and improving sensitivity (194). Additionally, curcuminoids can be quantified using UV-VIS if they absorb within the matrix or sample components. Curcumin exhibits maximum absorption at 425 nm (112, 190, 195).

Curcumin is also characterized using various spectroscopic techniques, including nuclear magnetic resonance, fluorescence, Fourier transform Raman spectroscopy, near-infrared spectroscopy, and Fourier transform infrared spectroscopy (196, 197).

Curcumin exists in three polymorphic forms: two orthorhombic and one monoclinic. Differential scanning calorimetry and X-ray diffraction analyses revealed that these polymorphs are monotropically related, with the monoclinic form being the most stable (198).

Electron paramagnetic resonance (EPR) spectroscopy is a non-invasive and efficient technique for examining materials containing unpaired electrons. According to Iravani and Soufi (199), EPR spectroscopy identifies different types of radicals and evaluates the antioxidative properties of compounds. Curcumin’s antioxidant properties have been assessed using EPR spectroscopy with free radicals such as 1,1-diphenyl2-picryl hydrazyl (DPPH), nitric oxide (NO·), hydroxyl (HO·), and superoxide (O_2_) (200, 201).

A study by Nikolic et al. (202) employed EPR spectroscopy to assess the antioxidant properties of low-energy nanoemulsions loaded with curcumin based on the stability of the tempol nitroxide free radical. The results demonstrated that the curcumin-containing nano-emulsion rapidly neutralized free radicals within the first 5 min (202).

## 11 Formulations of curcumin

Several curcumin formulations incorporating CP-01, piperine, and lecithin have been developed to improve absorption following oral administration compared to pure curcumin (203, 204). Innovative formulations with considerable potential include micelles, liposomes, phospholipid complexes, nanoparticles, cyclodextrins, emulsions, hydrogels, and phytosomes. These formulations enhance curcumin’s efficacy by increasing its circulation over extended periods, improving uptake and resisting metabolic processes, boosting absorption through the small intestine, and extending its plasma half-life (205, 206).

A study on healthy volunteers tested various formulations, including curcumin-containing phytosomes, CP-01, and hydrophilic carrier formulations containing cellulose derivatives and natural antioxidants (CHC) (144). These formulations were compared to a standard curcumin preparation. The results indicated that curcumin prepared using the CHC method achieved significantly higher blood curcuminoid levels than conventional preparations (204).

Cyclodextrins form molecular inclusion complexes with lipophilic substances, enhancing active components’ water solubility, dispersion, and absorption (20). One previous study examined the bioavailability of a curcumin formulation with γ-cyclodextrin. This formulation was compared to turmeric essential oils, curcumin phytosomes derived from rhizomes, and a standardized curcumin extract (20). The results showed that γ-cyclodextrin formulations significantly improved curcuminoid absorption in healthy individuals (20).

The coprecipitation method created a curcumin-β-cyclodextrin inclusion complex, significantly increasing curcumin’s water solubility from 0122 to 0.72100 mg/mL. Under simulated gastrointestinal conditions, the release of this inclusion complex was tested using standard poly (N-isopropyl acrylamide/sodium alginate) hydrogels cross-linked with nano-clay and N, N0-methylene bis(acrylamide) (BIS) (147). These nanocomposite hydrogels exhibited the minimum release-swelling ratio at a pH of 1.2 and the maximum at a pH of 6.8 (147). In nanocomposite hydrogels, increasing the nano-clay concentration resulted in a decrease in both the swelling coefficient and cumulative release. In contrast, with conventional hydrogels, the swelling ratio and cumulative release increased as the BIS ratio increased (207).

Kongkaneramit et al. (208) synthesized curcumin-containing liposomes using the polyol dilution technique. The lipid phase consisted of carbohydrates and hydrogenated phosphatidylcholine combined in a 9:1 molar ratio. Propylene glycol, glycerin, and polyethylene glycol 400 were used as polyol solvents. The curcumin content and liposome size were influenced by the type and concentration of polyol used, as well as the preparation temperature, which is a critical factor in liposome development (208).

Tai et al. (209) investigated curcumin’s stability and release properties in liposomes with varying hydrogenated phospholipid concentrations. They identified chitosan-coated liposomes as a potential drug delivery system. Cuomo et al. (210) further examined the efficacy of anionic liposomes and chitosan-coated liposomes containing curcumin. They tested the formulations *in vitro* by assessing the bioavailability of ingested curcumin. The positively charged surface of chitosan-coated liposomes enhanced curcumin’s overall bioavailability by facilitating its improved absorption in the small intestine (210).

A low-energy curcumin nano-emulsion was developed and converted into a nano-emulgel by incorporating cross-linked polyacrylic acid (Carbopol^®^ 934) as a gelling agent (151). This formulation was designed to enhance the solubility and absorption of curcumin when applied topically (151). In psoriatic mice, the nano-emulgel showed earlier and faster wound healing compared to both curcumin and betamethasone-17-valerate gel, indicating its potential for long-term psoriasis treatment (211). Additionally, curcumin nanoemulsions were shown to prevent postoperative tumor metastasis and recurrence effectively (212).

A thermosensitive hydrogel incorporating latanoprost and curcumin nanoparticles was recently developed as a formulation with a dual drug delivery system (153). This formulation demonstrated biocompatibility in both *in vitro* and *in vivo* studies, along with a delayed release profile (153). It also decreased inflammation and apoptosis while protecting trabecular mesh cells from oxidative damage (213). PLGA-based curcumin nanoparticles have shown enhanced oral and intravenous bioavailability (214). Saber-Moghaddem et al. (215) reported that administering oral nano-curcumin could significantly reduce recovery time in hospitalized patients with COVID-19. A curcumin-phospholipid complex, combining both curcumin and phospholipids, was employed for oral drug delivery to prevent metastases of breast and lung cancers (216).

Wang et al. (217) developed a curcumin-phospholipid complex with enhanced flow properties, solubility, and oral bioavailability, resulting in a high-performance formulation. Furthermore, polymer micelles composed of methoxy-poly(caprolactone)-poly(ethylene glycol) enabled the delayed release of curcumin (218). Liu et al. (219) developed the amylopectin–chitosan composite hydrogel (LRA–CS) for curcumin delivery and observed the dissolution characteristics of curcumin encapsulated in artificial stomachs and intestinal fluids (191). The results revealed that the LRA-CS hydrogel effectively maintained the stability of curcumin in the stomach and facilitated its controlled release in the small intestine (219). Furthermore, a hydrogel made of chitosan, nanocellulose, and a non-ionic surfactant was developed for curcumin delivery (220).

Transdermal administration of resveratrol and curcumin was achieved using cyclodextrin nanospongoid-based hydrogel (CDNS). This system significantly enhanced the *in vitro* release of curcumin and resveratrol by factors of 10 and 2.5, respectively, compared to their conventional forms (193). The combination of curcumin-CDNS and resveratrol-CDNS exhibited a synergistic cytotoxic effect on breast cancer MCF-7 cells lines. These formulations were integrated into a hydrogel base containing carbomer and propylene glycol (193). Adding CDNS to the hydrogel improved the photostability of resveratrol and curcumin approximately fivefold and sevenfold, respectively, compared to a hydrogel without CDNS. The use of a CDNS-hydrogel base notably increased the consumption of curcumin and resveratrol (221).

Curcumin was also incorporated into a hydrogel system based on oxidized cellulose and polyvinyl alcohol through a freezing process (222). Shefa et al. (222) demonstrated its efficacy in promoting spontaneous wound healing in *in vitro* rats studies. Sahin et al. (223) developed advanced ultrasol curcumin (AUC), a novel curcumin formulation with enhanced bioavailability and intestinal stability. AUC was demonstrated to improve the pathophysiology of experimentally induced osteoarthritis in rats significantly (222).

In clinical trials with healthy volunteers, the oral bioavailability of a novel curcumin formulation, Curene^®^, was compared to a formulation containing CP-01 and conventional curcuminoids 95%. Panda et al. (224) demonstrated that Curene^®^ had significantly better oral bioavailability (95%) than CP-01 and regular curcuminoids and was safe for use under trial conditions. Additionally, Longvida^®^ improved curcumin (LC) was studied for its anti-inflammatory properties in two-month-old wild-type and GFAP-IL6 mice. LC reduced inflammation and limited neurodegeneration and motor deficits in GFAP-IL6 mice (225).

Various commercial curcumin formulations exhibit bioavailability exceeding that of standard curcumin by more than 100-fold (21). These include products for oral administration and other formulations like Curcumin Rich, Liposomal Curcumin, Biomor, Dr. Mercola Curcumin Advanced, and Liposomal Curcumin Mango, which are available (205).

## 12 Health benefits and safety of curcumin

All three curcuminoids have shown potential therapeutic effects (226); however, studies have primarily focused on curcumin due to the growing evidence of its potential as a therapeutic agent in humans. A recent systematic review and meta-analysis of randomized-controlled trials (RCTs) using the Grading of Recommendations Assessment, Development, and Evaluation (GRADE) approach inferred that curcumin could improve inflammation and oxidative stress in adults ≥ 18 years of age across various health conditions (227).

The therapeutic potential of curcumin has become increasingly evident over the past several decades. Numerous studies have demonstrated its effectiveness against a range of cancers, including chemoresistant colon cancer cells, esophageal cancer, thyroid carcinoma, and skin cancer (228). Additionally, curcumin has shown strong anti-inflammatory properties (229). It exerts its anticancer effects by regulating various growth factors, protein kinases, inflammatory cytokines, and transcription factors, thereby inhibiting tumor proliferation and metastasis (228). Beyond its anticancer and antitumor activities, curcumin has also been found beneficial in treating various diseases such as respiratory tract infections, hepatic steatosis, skin photoaging, Parkinson’s disease, obesity, diabetes, HIV-associated diarrhea, and Alzheimer’s disease, primarily by inhibiting amyloid beta oligomer formation (230, 231).

Curcumin interacts with several targets within cancer pathways, notably protein kinases. It inhibits protein kinase C by forming hydrogen bonds with specific residues in the C1B subdomain and fits well within the binding pocket of glycogen synthase kinase-3β through interactions with key amino acids (232). Curcumin is also reported to act as a non-competitive inhibitor of phosphorylase kinase. Cyclin-dependent kinases (CDKs) are recognized as important cancer targets, with CDK1, CDK2, and CDK4/6 playing significant roles in cell cycle regulation. Disruptions in CDK activity are linked to cancer development (232).

Additional claims regarding curcumin’s antidiabetic, anticancer (233, 234), and hepatic benefits have been made. However, they are considered weak based on rigorous systematic reviews and meta-analyses (226, 235, 236). One systematic review linked curcumin’s potential antidiabetic effects to its anti-inflammatory and antioxidant properties (235).

Regarding safety, a recent literature review of double-blinded RCTs examined the safety and toxicity profiles of turmeric and curcumin in medical applications (237). Both turmeric and curcumin were safe for human use, particularly when taken orally. They were also considered safe in animal studies, showing non-mutagenic properties and safety during pregnancy. However, further studies in humans are recommended (237). Oral administration of curcumin at a dose of 6 g/day for 4–7 weeks was reported to be safe. However, minor gastrointestinal disturbances may occur (237).

Although curcumin is considered the primary curcuminoid for its therapeutic potential, recent studies have highlighted similar therapeutic potentials of several curcumin metabolites (238), such as CG, 2HC, 4HC, 6HC, and 8HC. These metabolites exhibit potential therapeutic effects, including antioxidative, anticancer, anti-inflammatory, and antiseptic properties, for various diseases, including liver disorders, neurological conditions, cancer, cardiovascular diseases, and lung diseases, as shown in multiple *in vitro* and *in vivo* studies on humans and mice (114, 116, 239).

However, the extent of therapeutic activity of curcumin metabolites compared to curcumin itself remains unclear. While 4HC and 6HC have been more extensively studied, research on 2HC and 8HC is relatively limited, necessitating further investigation into their therapeutic activities (114, 116, 239). Although *in vitro* and *in vivo* preclinical studies have demonstrated the therapeutic potential of these metabolites, further validation through animal models and subsequent clinical trials is required to provide robust evidence (206).

Clinical studies have consistently shown curcumin’s safety, tolerability, and effectiveness in managing various chronic human illnesses (19). Soleimani et al. (237) reported no adverse effects in humans after orally administering 6 g of curcumin per day for 4–7 weeks. Additionally, Greil et al. (240) investigated the safety, tolerability, and efficacy of liposomal curcumin (Lipocurc™) in patients with metastatic or locally advanced cancer. Their findings showed that a 300 mg/m^2^ dose of Lipocurc™ was the maximum dose that could be safely administered to individuals undergoing cancer therapy (240). Saghatelyan et al. (241) evaluated the combination of paclitaxel with intravenous curcumin infusion in patients with metastatic breast cancer. Following a 12-week treatment regimen, intravenous curcumin did not result in any significant adverse effects. Additionally, it did not negatively impact patients’ overall quality of life (241).

A cautionary note regarding potential herb-drug interactions is indeed warranted, particularly concerning curcumin with anticoagulants or chemotherapeutic agents.

### 12.1 Curcumin and anticoagulants

Curcumin, the bioactive component of turmeric, has demonstrated antiplatelet and anticoagulant properties in numerous studies. This indicates its capacity to impede blood coagulation. The concomitant use of anticoagulant drugs (including warfarin, clopidogrel, aspirin, or dabigatran) elevates the risk of bleeding, bruising, and hematoma development (242). The combined effect can lead to an amplified anticoagulant effect, potentially pushing the patient beyond their therapeutic range and increasing the likelihood of hemorrhagic complications (242).

### 12.2 Curcumin and chemotherapeutic agents

The interaction between curcumin and chemotherapeutic agents is more complex and can be either beneficial or detrimental, depending on the specific chemotherapy drug and cancer type (243).

#### 12.2.1 Potential synergistic effects

In some preclinical studies, curcumin has shown the ability to enhance the efficacy of certain chemotherapeutic agents (e.g., paclitaxel, gemcitabine, cisplatin) by sensitizing cancer cells to the drugs, inhibiting drug resistance, and promoting apoptosis. This could potentially allow for lower doses of chemotherapy or improve treatment outcomes (243).

#### 12.2.2 Potential antagonistic effects

Conversely, curcumin has been shown to interfere with the action of other chemotherapeutic drugs (e.g., cyclophosphamide, doxorubicin) by activating certain detoxification pathways (like ABC transporters) that pump the drugs out of cancer cells, thereby reducing their effectiveness (244). There are also concerns that curcumin’s antioxidant properties could, in some contexts, protect cancer cells from the oxidative damage induced by certain chemotherapies (244).

### 12.3 General cautionary points for herb-drug interactions

The combination of herbs and drugs can lead to an exaggeration of either the drug’s or the herb’s effects, or introduce new, unpredictable adverse reactions. Herbs can sometimes accelerate the metabolism or excretion of drugs, leading to sub-therapeutic drug levels and reduced treatment effectiveness (245, 246).

Herbs can influence drug absorption, distribution, metabolism (especially via cytochrome P450 enzymes), and excretion, leading to altered drug concentrations in the body. Herbal products often lack the rigorous standardization and regulation of pharmaceutical drugs, leading to variability in their active compound content and potential contamination (246, 247).

While *in vitro* and animal studies exist, robust human clinical trials on many herb-drug interactions are often limited, making it difficult to predict precise effects in patients (245–247).

Patients must inform their healthcare providers (doctors, pharmacists, nurses) about all herbal supplements, traditional medicines, and dietary supplements they are taking, even if they seem innocuous. Healthcare professionals should proactively inquire about such use (245, 247). This allows for identifying potential interactions and assessing the patient’s risk factors and modifying drug dosages if necessary. Continuous monitoring for adverse effects or altered drug efficacy is essential (246). Moreover, it is essential to inform patients about the significance of avoiding self-medication with herbal remedies while undergoing conventional pharmacological therapy, as well as the possible risks of interactions (245–247).

In conclusion, while herbs such as curcumin offer potential health benefits, their interaction with prescription medications can have significant clinical implications, ranging from increased toxicity to reduced therapeutic efficacy (245). A cautious and informed approach, with open communication between patients and healthcare providers, is paramount to ensuring patient safety and optimal treatment outcomes (246, 247).

## 13 Biological activities of curcumin

The therapeutic properties of turmeric are quite diverse, primarily due to the physiological benefits attributed to curcumin (248). In animal studies, oral administration of curcumin has demonstrated significant anti-inflammatory and antiparasitic efficacy (249, 250). Additionally, *in vitro* studies have shown its anti-inflammatory, antispasmodic, anti-carcinogenic, and gastrointestinal protective properties (248–250).

**Table 2** illustrates the biological activity, mechanisms, and therapeutic uses of curcumin and its derivatives.

**Table 2:** Biological activity, mechanisms, and clinical applications of curcumin and its derivatives.

Compound	Biological Activity	Mechanism of Action	Clinical Application	References
Curcumin	Anti-inflammatory	Inhibits NF-κB, COX-2, and TNF-α	Arthritis, inflammatory diseases	(251, 252)
Curcumin	Antioxidant	Scavenges ROS, upregulates SOD and catalase	Neuroprotection, aging	(253)
Curcumin	Anticancer	Induces apoptosis (p53, Bax), inhibits Akt/mTOR	Colorectal, breast, prostate cancer	(254, 255)
Demethoxycurcumin	Antiviral	Blocks viral entry (e.g., SARS-CoV-2 spike protein)	COVID-19 (under investigation)	(256, 257)
Demethoxycurcumin	Neuroprotective	Reduces Aβ plaque aggregation	Alzheimer's disease	(258, 259)
Bisdemethoxycurcumin	Antidiabetic	Enhances GLUT4 translocation, AMPK activation	Type 2 diabetes	(260, 261)
Tetrahydrocurcumin	Anti-aging	Activates Nrf2/ARE pathway	Skin aging, oxidative stress	(262, 263)
	Hepatoprotective	Reduces lipid peroxidation, CYP2E1 inhibition	Liver fibrosis, NAFLD	(264, 265)
Curcumin-glucoside	Improved bioavailability	Enhanced water solubility	Drug delivery enhancement	(266)
Curcumin-PLGA NPs	Anticancer (targeted)	Enhanced tumor accumulation, pH-sensitive release	Pancreatic cancer therapy	(267, 268)
Curcumin-cyclodextrin	Anti-ulcer	Reduces gastric acid secretion, enhances mucus production	Peptic ulcer disease	(269, 270)
Curcumin-metal complexes (e.g., Cu, Zn)	Antimicrobial	Disrupts bacterial cell membranes, generates ROS	Antibacterial/antifungal infections	(271)
Curcumin-phospholipid complex	Wound healing	Stimulates collagen synthesis, angiogenesis	Diabetic wounds burns	(272, 273)
Curcumin-loaded liposomes	Cardioprotective	Reduces myocardial oxidative stress, inhibits apoptosis	Ischemic heart disease	(274, 275)
Curcumin analogs (EF24)	Anticancer (potent)	Inhibits STAT3, NF-κB, and Wnt/β-catenin	Ovarian lung cancers	(276, 277)
Curcumin-polymeric micelles	Anti-arthritic	Suppresses IL-6, IL-1β	Rheumatoid arthritis	(278, 279)
Curcumin-quercetin hybrid	Synergistic antioxidant	Enhances radical scavenging, metal chelation	Neurodegenerative diseases	(280, 281)
Curcumin-selenium nanoparticles	Antidiabetic	Mimics glutathione peroxidase, improves insulin sensitivity	Diabetes mellitus	(282)
Curcumin-resveratrol hybrid	Anti-aging	Activates SIRT1, inhibits MMP-1	Skin photoaging	(283, 284)
Curcumin-loaded exosomes	Targeted drug delivery	Enhanced blood-brain barrier (BBB) penetration	Glioblastoma, neurodegenerative disorders	(285, 286)

NF-κB, Nuclear factor kappa-light-chain-enhancer of activated B cells; COX-2, Cyclooxygenase-2; TNF-α, Tumor necrosis factor-alpha; ROS, Reactive oxygen species; SOD, Superoxide dismutase; p53, Tumor protein p53; Bax, Bcl-2-associated X protein; Akt/mTOR, Protein kinase B/mammalian target of rapamycin; SARS-CoV-2, Severe acute respiratory syndrome Coronavirus 2; Aβ, Amyloid-beta; GLUT4, Glucose transporter type 4; AMPK, AMP-activated protein kinase; Nrf2/ARE, Nuclear factor erythroid 2-related factor 2/Antioxidant response element; CYP2E1, Cytochrome P450 2E1; NAFLD, Non-alcoholic fatty liver disease; PLGA NPs, Poly(lactic-co-glycolic acid) nanoparticles; Cu, Copper; Zn, Zinc; STAT3, Signal transducer and activator of transcription 3; Wnt/β-catenin, Wingless/integrated and beta-catenin; IL-6, Interleukin 6; IL-1β, Interleukin 1 beta; MMP-1, Matrix metalloproteinase-1; SIRT1, Sirtuin 1; BBB, Blood-brain barrier.

### 13.1 Wound healing activity of curcumin

Curcumin plays a significant role in enhancing the wound-healing process, which comprises four stages: coagulation, inflammation, proliferation, and remodeling (287). In the initial stage, curcumin promotes programmed cell death of inflammatory cells, hinders the transcription factor NF-κB, decreases cytokine production (tumor necrosis factor-alpha (TNF-α) and interleukin (IL)-1), and lowers reactive oxygen species (ROS) levels (287). These effects enhance antioxidant enzyme synthesis, decrease inflammation, and accelerate the resolution of the inflammatory phase. During the proliferative phase, curcumin promotes collagen deposition, fibroblast migration, granulation tissue development, and re-epithelialization. In the final phase, curcumin boosts TGF-β synthesis, promoting wound contraction and fibroblast development (287).

Various topical formulations, including films, emulsions, fibers, hydrogels, and nano- formulations, have been created to deliver curcumin selectively to wound sites (287–289). Zakerikhoob et al. (288) developed sodium alginate-g-poly (N-isopropyl acrylamide) (Alg-pNIPAM) loaded with curcumin, a thermosensitive hydrogel, for *in vivo* wound dressing. Studies demonstrated that the Alg-pNIPAM formulation accelerated collagen production, epithelial cell regrowth, and wound contraction. The formulation also exhibited superior anti-inflammatory effects compared to free curcumin solutions. The thermosensitive formulation combines curcumin’s antioxidant and anti-inflammatory properties with the moisture-retaining capability of alginate to expedite the wound healing process (288).

### 13.2 Anti-inflammatory activity of curcumin

Inflammation plays a pivotal role in the development of many health conditions, including cardiovascular diseases (290), cancer (291), diabetes (292), and neurodegenerative disorders (293). TNF-α is a key mediator in the signal transduction pathways linked to inflammatory diseases, along with other inflammatory mediators. Tak and Firestein (294) identified NF-κB as a potential therapeutic target due to its involvement in these disorders. ROS also contributes significantly to inflammation in various illnesses by modulating transcription factors like NF-κB and activator protein 1 (AP-1) through nuclear histone acetylation and deacetylation (295). Dysregulation of COX-2 and iNOS has been implicated in inflammatory diseases and the pathogenesis of several cancers (296).

Several studies have demonstrated significant anti-inflammatory effects of turmeric, particularly its active component curcumin, through the inhibition of TNF-α (297–299). A systematic review and meta-analysis by Daily et al. (300) reported that curcumin supplementation could effectively decrease inflammatory markers, including C-reactive protein (CRP) and IL-6, in both healthy individuals and those with chronic diseases (300, 301).

Additionally, curcumin has been shown to suppress the production of inflammatory cytokines such as interferon, TNF-α, IL-1, IL-6, and IL-8 (302, 303). A preclinical study by Banik et al. (296) explored curcumin analogs like DM1, which inhibits iNOS and COX2, further highlighting curcumin’s anti-inflammatory potential (304).

Curcumin, often combined with rutin, decreases COX-2 levels and tumor-related inflammation in HPV16-expressing mice (305). In preclinical animal models of invasive pneumonia, curcumin modulates pro- and anti-inflammatory factors (COX-2, IL-6, IL-8, and IL-10), induces apoptosis in polymorphonuclear neutrophilic cells, and mitigates ROS damage (235). Moreover, curcumin has demonstrated efficacy in the treatment of acute lung injury and fatal acute respiratory distress syndrome caused by human coronaviruses in multiple trials (306).

### 13.3 Immunomodulatory mechanisms and novel applications

Curcumin shifts macrophages from pro-inflammatory (M1) to anti-inflammatory (M2) phenotypes by suppressing TLR4/NF-κB and JAK-STAT pathways, reducing IL-6, TNF-α, and nitric oxide production. This is being explored in rheumatoid arthritis and neuroinflammatory diseases (307, 308). Also, It inhibits Th17 differentiation (reducing IL-17) while promoting regulatory T-cell (Treg) activity via TGF-β1 upregulation. This rebalances immune responses in autoimmune conditions like systemic lupus erythematosus (SLE) and psoriasis (43, 309). Furthermore, curcumin blocks dendritic cell (DC) maturation and antigen presentation, dampening adaptive immune activation. This is leveraged in colitis models to induce intestinal Treg differentiation (310, 311).

Curcumin suppresses NF-κB, MAPK, and JAK/STAT signaling, downregulating pro-inflammatory cytokines (IL-1β, IL-6, TNF-α) while enhancing anti-inflammatory IL-10. It also inhibits ROS-generating enzymes (COX-2, iNOS), mitigating oxidative stress (312–314). Also, It reduces PD-1 expression on exhausted T cells, potentially reversing age-related immune decline and improving vaccine responses (315).

Curcumin also enhances NK cell activity and CD8+ T-cell cytotoxicity against tumors. Derivatives like FLLL32 degrade STAT3, suppressing tumor angiogenesis (VEGF) and metastasis (MMP-2/9) in preclinical osteosarcoma models (316–318).

### 13.4 Antioxidant activity of curcumin

Oxidative stress arises from an imbalance between endogenous antioxidants and ROS naturally produced in the human body (237). ROS generated during normal cellular processes, such as respiration, include singlet oxygen (¹O_2_), hydroxyl radicals (HO·), superoxide radicals (O_2_), and hydrogen peroxide (H_2_O_2_). Excess ROS can oxidize biological components, leading to tissue damage (237). The human body uses antioxidant defense systems, including superoxide dismutase (SOD), catalase (CAT), and reduced glutathione to prevent ROS-induced harm (319). Sankar et al. (320) suggested that both free and encapsulated curcumin can indirectly enhance the activity of antioxidant enzymes such as glutathione reductase, SOD, and CAT (320). Curcumin also demonstrates synergistic antioxidant effects when combined with other antioxidants (321).

Kanwal et al. (322) stated that curcumin nanoparticles of different sizes provide increased surface area to expose the functional phenolic groups more effectively, which makes them able to display their free radical scavenging ability effectively. Curcumin exhibits potential biological activities to fight against chronic diseases and can act on several molecular pathways (322). However, the antioxidant feature of curcumin is considered the most important one. Oxidative stress is a result of an imbalance between the elimination and production of ROS and can be related to many chronic diseases and the aging process (323). The properties of curcumin responsible for the removal of reactive nitrogen and oxygen, regulation of different enzymes, and metal chelation are because of the action of curcumin on markers of oxidative stress. This proves that curcumin acts as a potential antioxidant (324).

Curcumin contains various functional groups, and the phenolic functional groups trap electrons to deter the production of H_2_O_2_ and scavenge the superoxide radicals, and the β-dike to group produces metal-ligand complexation and carbon-to-carbon double bonds. Thus, the molecule shows some unique antioxidant characteristics (325). Some researchers reported the antioxidant activities of curcumin versus oxidative stress caused by diabetes mellitus. The study was performed on cochlear fibroblasts in rat models of diabetes mellitus, and the authors concluded that an increased expression of superoxide dismutase causes curcumin to confer antioxidant protection (285).

The antiradical power of an antioxidant can be determined with the DPPH method by measuring a decrease in absorbance of DPPH at 515 nm. An antioxidant scavenges the DPPH by donating hydrogen to form a stable DPPH molecule, and the absorbance decreases as a result. The molecules, in the radical form, give an absorbance at 515 nm, which disappears after acceptance of hydrogen or an electron from an antioxidant compound to become a diamagnetic stable molecule (326).

Studies on turmeric leaves further demonstrate the antioxidant properties attributed to its bioactive compounds. Braga et al. (327) highlighted curcumin, total phenolic compounds, and flavonoids as some bioactive substances that contribute to the antioxidant capacity of the leaves. Over the last two decades, many studies have examined the underlying mechanisms of curcumin’s antioxidant properties and its ability to scavenge free radicals, thus reducing cellular oxidative damage (27). Additional tests by Kuncha et al. (328) showed that curcumin can decrease oxidative stress and inflammation in the liver by augmenting antioxidant enzyme activity (328).

Similarly, Jagetia et al. (325) reported that curcumin can prevent stress-induced oxidative damage in the liver, kidneys, and brain of rats. It also shields cells from oxidative damage caused by radiation by reducing ROS production and lipid peroxidation (27). However, despite these studies, the precise antioxidant mechanism of curcumin remains debated. The key point of contention is whether the activity stems from the enolic hydrogen, the phenolic hydrogen, or the central methylenic hydrogen of the heptadiene moiety (329).

Jovanovic et al. (330) demonstrated that curcumin acts as a potent hydrogen atom donor, primarily through the central methylenic group rather than the phenolic group (330). This finding contrasts with the traditional view of curcumin as a phenolic chain-breaking antioxidant donating hydrogen atoms from the phenolic group (331).

Recent studies have explored several curcumin analogs to evaluate their antioxidant efficacy (246, 247). Even with their lack of phenolic hydrogen, these analogs exhibited antioxidant activity comparable to curcumin. Oglah et al. (332) suggested that their ability to donate enolic hydrogen may be a significant contributor to their antioxidant properties (332). Other studies have highlighted the necessity of curcumin’s phenolic hydroxyl group for scavenging free radicals, with the antioxidant effect further enhanced by the addition of a methoxy group to this hydroxyl group (333).

Numerous curcumin derivatives have been synthesized over the last two decades to develop molecules with enhanced antioxidant activity. Shang et al. (334) assessed the antioxidant potential of three series of curcumin derivatives. Compounds containing O-diphenoxyl- and O-dimethoxy-phenoxyl groups exhibited significantly greater antioxidant activity than those without these moieties. Their findings also showed that the antioxidant activity depends on the presence of a seven-carbon spacer; reducing the spacer to five carbons significantly diminished the activity. Additionally, they proposed that lipophilicity, which is enhanced by increasing the amount of carbon atoms, influences antioxidant efficacy (334).

Curcumin has been shown to boost enzymatic antioxidant activity by increasing the levels of enzymes such as SOD, CAT, glutathione peroxidase, and methionine sulfoxide reductase (335). For example, curcumin protects diabetic rats from oxidative stress by upregulating SOD expression in cochlear fibroblasts (336). Comparatively, curcumin demonstrated similar antioxidant activity to ascorbic acid in the DPPH radical scavenging test. At a concentration of 0.1 mM, curcumin achieved a 69% free radical elimination rate, compared to 62% for ascorbic acid, indicating no significant difference between the two antioxidants (337).

Curcumin appears to be especially effective at scavenging smaller oxidative molecules, including H_2_O_2_, HO•, and ROO•. Chen et al. (338) demonstrated that curcumin is a potent antioxidant capable of protecting cell cytoplasm from ROS. Stabilized formulations of curcumin with specific carriers have been developed to enhance its stability and antioxidant potential under certain stressful conditions (339, 340).

**Figure 6:**
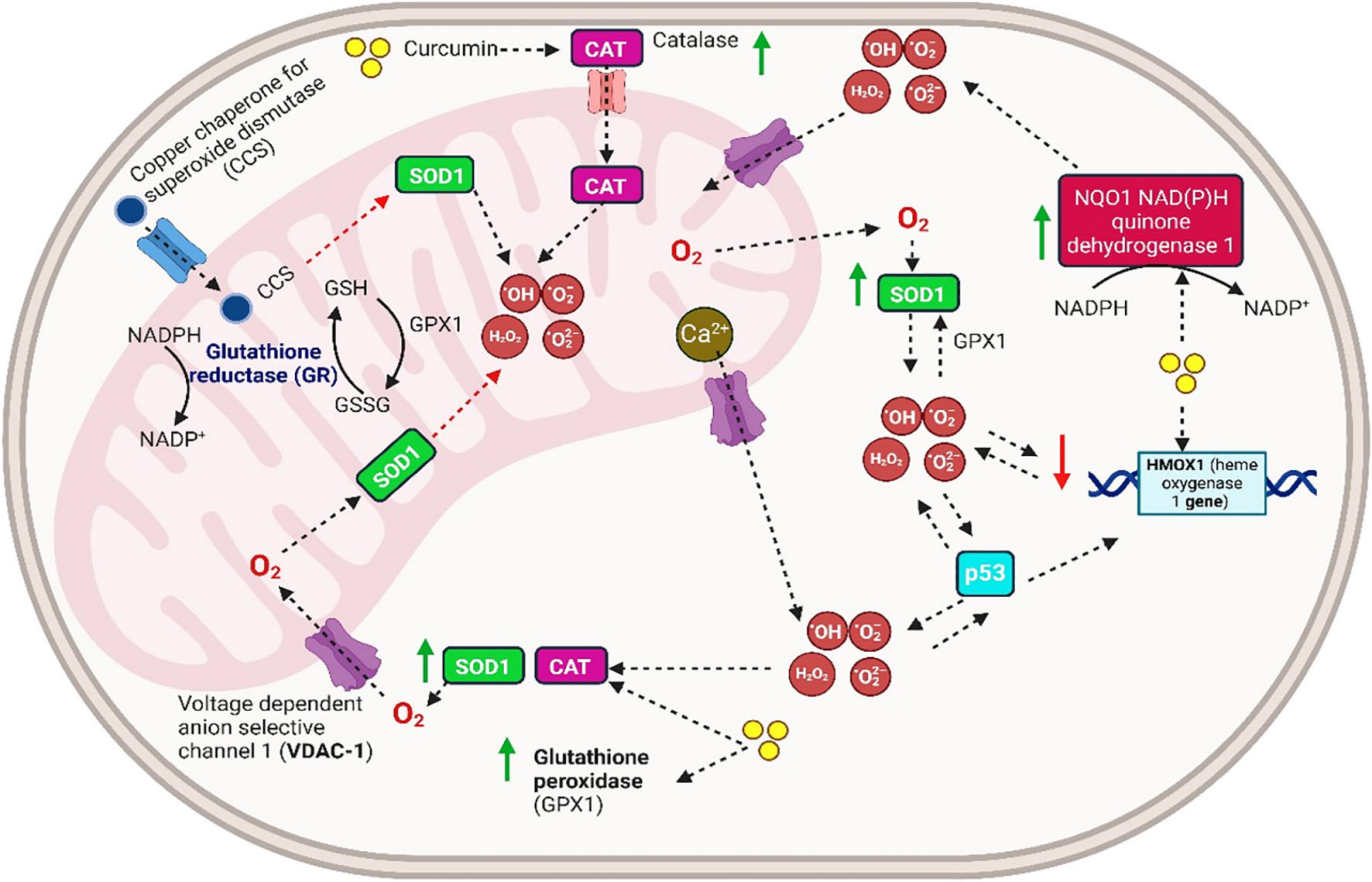
Antioxidant properties of curcumin.

Curcumin not only neutralizes harmful free radicals but also improves the efficacy of other antioxidants. While these findings are promising, further clinical studies in humans are required to validate the full extent of curcumin’s antioxidant benefits. The antioxidant capabilities of curcumin are demonstrated in **Figure 6**.

### 13.5 Antibacterial activity of curcumin

Curcumin’s antibacterial properties were first reported by Schraufstätter and Berntet (341), and over the past 70 years, extensive research has confirmed its broad-spectrum activity against various microorganisms. Curcumin exhibits notable antibacterial effects against Gram-positive and Gram-negative bacteria (342, 343).

The global rise in antibiotic resistance and the associated risk of treatment failures have intensified the search for novel antimicrobial agents (344–346). For instance, *Staphylococcus aureus* infections, particularly methicillin-resistant *S. aureus* (MRSA), pose significant challenges in low-resource settings and hospital environments (347). These infections have contributed to increased morbidity and mortality rates over time (348). Recent studies indicate that curcumin shows potent antibacterial activity against both MRSA and methicillin-sensitive *S. aureus* (MSSA) (349, 350).

Curcumin not only displays intrinsic antibacterial properties but also enhances the efficacy of ampicillin, ciprofloxacin, norfloxacin, gentamicin, and amikacin when used in combination against *S. aureus* (351). Furthermore, it has been shown to inhibit the growth of various harmful bacteria, including *Enterococcus faecalis, Pseudomonas aeruginosa, Escherichia coli*, and *Klebsiella pneumoniae* (352). Its mechanism of action involves disrupting bacterial membranes in both Gram-positive bacteria (*S. aureus* and *E. faecalis*) and Gram-negative bacteria (*E. coli* and *P. aeruginosa*) (353).

Curcumin exerts its antibacterial effects through several pathways: it inhibits bacterial growth, reduces the production of biofilms and virulence factors, blocks bacterial adhesion to host cells, and suppresses the generation of oxidative compounds (246). Advances in curcumin analogs have further enhanced its antibacterial potential. For example, the curcumin analog CA2, which replaces guaiacol rings with halogenated coumarin rings, demonstrated greater water solubility and stronger antibacterial activity than curcumin against pathogens, such as *Haemophilus influenzae, E. coli, P. aeruginosa*, and *K. pneumonia* (332).

**Figure 7:**
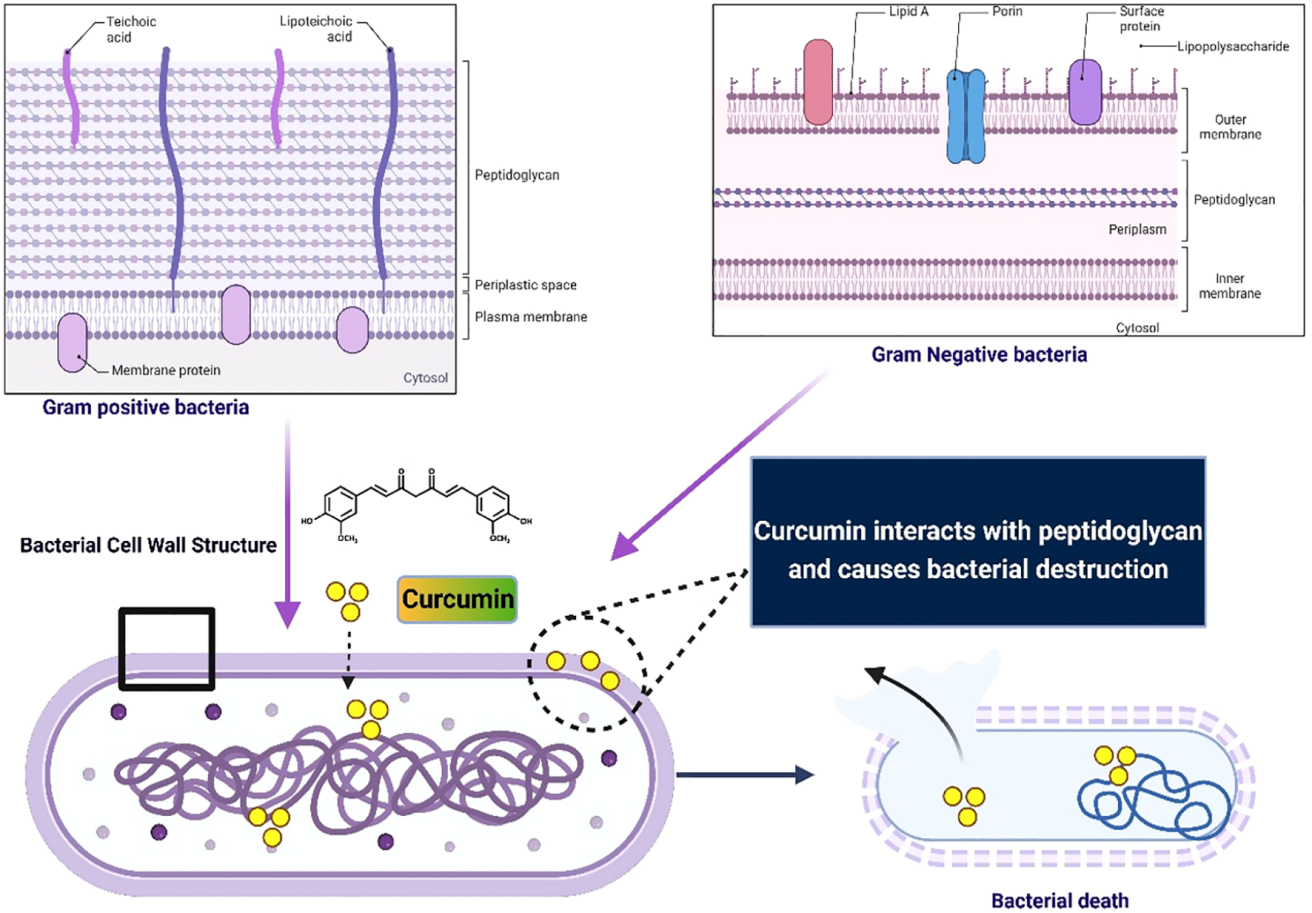
Antibacterial characteristics of curcumin.

Curcumin’s activity extends to light-dependent mechanisms. Under blue light, it acts as a photosensitizer, inducing phototoxicity to inhibit bacterial growth (354). A study by Adamczeck et al. (355) evaluated curcumin’s antibacterial efficacy against over 100 pathogens from 19 species using the broth microdilution method. Results showed that Gram-positive bacteria are generally more susceptible than Gram-negative bacteria. Notably, curcumin displayed potent activity against *Streptococcus pyogenes* (MIC = 31.25 µg/mL), methicillin-susceptible *S. aureus* (MIC = 250 µg/mL), *Acinetobacter lwoffii* (MIC = 250 µg/mL), and certain isolates of *P. aeruginosa* and *E. faecalis* ((MIC = 62.5 µg/mL) (355). However, curcumin had limited efficacy against clinical isolates of *Candida* species (305). Despite its selective activity, curcumin holds promise as a potential antibacterial agent, particularly against resistant bacterial strains. These findings highlight its potential to complement existing treatments and address the growing challenge of antibiotic resistance. The antibacterial properties of curcumin are outlined in **Table 3** and **Figure 7**.

**Table 3:** Antibacterial activity of curcumin.

Bacteria	Action mechanism	References
*Staphylococcus aureus*	Inhibition of biofilm formation, disruption of cell membrane integrity, interference with the FtsZ protein involved in cell division, and inhibition of growth.	(356, 357)
*Escherichia coli*	Disruption of the bacterial cell membrane and increased membrane permeability leads to leakage of cellular contents and inhibition of ATPase activity.	(356, 358)
*Pseudomonas aeruginosa*	Impairs quorum sensing mechanisms, inhibits biofilm formation, disrupts membrane integrity, and reduces the expression of virulence factors.	(359, 360)
*Salmonella typhimurium*	Induction of oxidative stress, disrupts the bacterial cell membrane, increases reactive oxygen species generation, and causes bacterial cell death.	(361)
*Listeria monocytogenes*	Inhibits biofilm formation, disrupts membrane integrity, and causes loss of membrane potential and leakage of intracellular ions.	(362)
*Bacillus subtilis*	Affects membrane structure, inhibits spore formation, reduces intracellular ATP levels, and interferes with bacterial cell division.	(363)
*Mycobacterium tuberculosis*	Modulates macrophage responses, increases autophagy, induces apoptosis in infected cells, and enhances the efficacy of conventional antimycobacterial drugs.	(364, 365)
*Klebsiella pneumoniae*	Disrupts cell membrane integrity, inhibits biofilm formation, and reduces bacterial virulence by affecting gene expression linked to quorum sensing.	(365, 366)
*Enterococcus faecalis*	Inhibits bacterial virulence factors, disrupts biofilm formation, and affects the integrity of the bacterial membrane and cell wall.	(367)
*Helicobacter pylori*	Inhibits urease activity, suppresses bacterial adhesion to gastric mucosa, disrupts membrane function, and induces bacterial cell death via oxidative stress.	(368)
*Streptococcus mutans*	It inhibits glucosyltransferase activity, disrupts bacterial adhesion and biofilm formation, and affects acid production, reducing cariogenic potential.	(369)
*Acinetobacter baumannii*	Inhibits biofilm formation, disrupts membrane permeability, induces reactive oxygen species production, and enhances susceptibility to conventional antibiotics.	(370)
*Vibrio cholerae*	Inhibits bacterial motility and quorum sensing, disrupts membrane integrity, and reduces the production of virulence factors like cholera toxin.	(356, 364, 365)
*Streptococcus pneumoniae*	Disrupts biofilm formation, interferes with bacterial autolysins, and affects the bacterial membrane, leading to growth inhibition.	(371, 372)
*Clostridium difficile*	Inhibits spore germination, disrupts bacterial cell wall integrity, and enhances the activity of other antimicrobial agents.	(373)
*Neisseria gonorrhoeae*	Disrupts bacterial membranes, induces oxidative stress, and inhibits biofilm formation, thereby reducing virulence and pathogenicity.	(374, 375)

### 13.6 Antifungal activity of curcumin

In recent decades, fungal infections have significantly increased worldwide, with *Candida* species being among the most common culprits (376). Among these, *Candida albicans* stands out as the most virulent and is recognized as a primary fungal pathogen in humans (377, 378). Studies have demonstrated that curcumin not only effectively inhibits the growth of *C. albicans* isolates but also enhances the efficacy of fluconazole, thereby reducing the pathogen’s resistance to this widely used antifungal agent (379).

In addition, two curcumin derivatives—1,7-bis (3,4,5- trimethoxy phenyl)-1,6-heptadiene-3,5-dione (L1) and 1,7-di(9-anthracenyl)-1,6-heptadiene-3,5-dione (L2) were synthesized and evaluated for their antifungal activity against the genera *Aspergillus*, *Penicillium*, and *Alternaria*. Both derivatives displayed inhibitory effects on the tested cultures, with L1 demonstrating superior antifungal activity compared to L2 (380). These findings suggest curcumin’s potential as a versatile antifungal agent in its natural form or as a chemically modified derivative. The effectiveness of curcumin as an antifungal agent is presented in **Table 4**.

**Table 4:** Antifungal activity of curcumin.

Fungi	Mechanisms of action	References
*Candida albicans*	Inhibition of biofilm formation, disruption of cell membrane integrity by increasing permeability, induction of reactive oxygen species production, and inhibition of hyphal growth.	(381–384)
*Aspergillus fumigatus*	Inhibits spore germination, disrupts membrane integrity, induces reactive oxygen species production, and interferes with the fungal cell wall biosynthesis.	(385, 386)
*Cryptococcus neoformans*	Disrupts cell membrane function, and inhibits melanin production, reducing fungal virulence and enhancing reactive oxygen species production.	(383, 387)
*Trichophyton rubrum*	Disrupts fungal cell wall synthesis, inhibits biofilm formation, and induces oxidative stress, leading to fungal cell death.	(388, 389)
*Fusarium oxysporum*	Induces oxidative stress, inhibits conidial germination, disrupts membrane integrity, and interferes with ergosterol biosynthesis.	(390)
*Aspergillus niger*	Disrupts cell membrane, increases permeability, induces reactive oxygen species accumulation, and inhibits spore germination.	(391, 392)
*Malassezia furfur*	Inhibits fungal biofilm formation, disrupts membrane integrity, and interferes with lipid metabolism necessary for fungal survival.	(393)
*Penicillium expansum*	Disrupts fungal spore germination, induces oxidative stress, inhibits cell wall synthesis, and alters membrane permeability.	(394, 395)
*Alternaria alternata*	Inhibits fungal growth, induces oxidative stress, interferes with fungal sporulation, and disrupts membrane function.	(396, 397)
*Candida glabrata*	Inhibits biofilm formation, disrupts mitochondrial function, induces oxidative stress, and interferes with ergosterol biosynthesis.	(381)
*Microsporum canis*	Disrupts membrane integrity, inhibits biofilm formation, and induces reactive oxygen species production, leading to fungal cell death.	(398, 399)
*Rhizopus oryzae*	Inhibits spore germination, disrupts cell wall integrity, induces oxidative stress, and alters fungal morphology.	(399, 400)
*Botrytis cinerea*	Inhibits fungal growth, induces oxidative damage, interferes with spore germination, and disrupts membrane integrity.	(401, 402)
*Candida krusei*	Disrupts mitochondrial function, interferes with fungal biofilm formation, and alters ergosterol biosynthesis.	(384, 386, 403)
*Paracoccidioides brasiliensis*	Disrupts membrane integrity, induces oxidative stress, inhibits cell wall biosynthesis, and induces programmed cell death.	(404, 405)
*Sporothrix schenckil*	Disrupts fungal membrane, interferes with cell wall biosynthesis, induces oxidative stress, and inhibits biofilm formation.	(386, 406)
*Saccharomyces cerevisiae*	Disrupts mitochondrial function, induces reactive oxygen species production, and interferes with cell wall biosynthesis and cell cycle progression.	(11, 407)

### 13.7 Antiviral activity of curcumin

*C. longa*, particularly its bioactive compound curcumin, has been extensively studied for its potential antiviral properties (311). Research indicates that curcumin can inhibit the replication of various viruses and modulate immune responses. For example, combining curcumin with acyclovir significantly enhances the antiviral effect against the herpes simplex virus compared to acyclovir alone (408). Curcumin’s antiviral mechanisms include suppressing viral replication and targeting critical cellular signaling pathways such as the phosphatidylinositol 3-kinase/protein kinase B (PI3K/Akt and NF-κB (409).

Studies have shown curcumin’s broad-spectrum antiviral efficacy against DNA and RNA viruses (410). For instance, Jeong et al. (411) demonstrated that curcumin pretreatment in fathead minnow cells effectively blocked the early stage of viral hemorrhagic septicemia virus (VHSV) infection. This was attributed to curcumin’s ability to alter the F-actin/G-actin ratio, impeding viral entry into cells (314). In addition, curcumin has shown significant inhibitory effects on herpes simplex virus type 2 (HSV-2) and HIV-1 in vaginal epithelial cells and T lymphocytes, respectively (412).

Curcumin’s impact on various viral diseases is partly due to its inhibition of apurinic/apyrimidinic endonuclease 1 (APE1’s redox function, which influences numerous genes. Li et al. (413) reported that curcumin efficiently blocks the replication of Kaposi’s sarcoma-associated herpesvirus and inhibits angiogenesis and cellular invasion. Moreover, curcumin exhibits antiviral activity against several other viruses, including chikungunya virus, Zika virus, dengue virus, hepatitis C virus, coxsackievirus, human papillomavirus, and SARS-CoV-2 (414, 415).

In the context of COVID-19, curcumin has garnered attention due to its multifaceted therapeutic properties, including its ability to reduce inflammation, modulate the immune system, alleviate pain, and protect lung tissue (416–418). Research suggests that curcumin may interact with Angiotensin 2 (ACE2) or spike proteins involved in the SARS-CoV-2 signaling pathway, thereby disrupting viral processes. Curcumin also inhibits key signaling pathways and transcription factors associated with viral inflammation, such as NF-κB, signal transducer and activator of transcription 3 (STAT-3), Vnt/b-catenin, nuclear factor E2-related factor (Nrf2), and p38/MAPK (419, 420).

A study by Valizadeh et al. (421) demonstrated the potential of nano curcumin in modulating inflammatory responses in COVID-19 patients. Real-time polymerase chain reaction (PCR) and the enzyme-linked immunosorbent assay (ELISA) analyses revealed significant reductions in mRNA levels and cytokine secretion of IL-1β and IL-6, respectively, following nano-curcumin administration (324). These findings highlight curcumin’s potential as a novel therapeutic agent for managing COVID-19 by reducing inflammation and improving immune regulation (421).

Curcumin has shown the potential to enhance the immune response to COVID-19 vaccination (422). Several studies indicated that curcumin supplementation can significantly increase SARS-CoV-2 antibody production in vaccinated individuals (423, 424). It also has dose-dependent immunoediting potential, influencing T-cell production and enhancing the function of effector T-cells while reducing regulatory T-cells (316). Curcumin may prevent critical COVID-19 by blocking ACE2 production (necessary for viral entry) and stimulating anticoagulation and fibrinolysis (425). It has also shown efficacy in preventing severe pneumonia, potentially by acting on the IL-6 trans signal and HM-GB1 system (426).

Further investigation into the specific molecular pathways and cellular targets by which curcumin exerts its immunomodulatory and antiviral actions is necessary. It is also essential to conduct more rigorous and large-scale clinical trials to test curcumin’s safety and efficacy, determine appropriate doses, and assess long-term results for specific immunological disorders and infectious diseases, such as COVID-19, across diverse human populations. The antiviral properties of curcumin are displayed in **Table 5**.

**Table 5:** Antiviral activity of curcumin.

Viruses	Mechanisms of action	References
Hepatitis C virus (HCV)	Inhibits viral entry, replication, and translation by targeting viral protease activity and affecting cellular lipid metabolism. It also inhibits NS3 protease.	(427, 428)
HIV-1 (Human immunodeficiency virus)	Inhibits viral replication by blocking HIV-1 integrase, protease, and reverse transcriptase activities and downregulates co-receptors (CCR5 and CXCR4) on host cells.	(429, 430)
Zika virus	Inhibits viral replication by disrupting viral protease function and interfering with entry into host cells. It also reduces viral load and pathogenesis.	(431, 432)
Influenza A virus	Blocks hemagglutinin-mediated viral entry, inhibits neuraminidase activity, and interferes with viral RNA replication.	(409, 433)
Herpes simplex virus type 1 (HSV-1)	Prevents viral entry and replication by inhibiting glycoproteins (gB and gC), disrupts viral envelope, and inhibits viral DNA polymerase activity|.	(434, 435)
SARS-CoV-2 (COVID-19)	Inhibits viral spike protein binding to ACE2 receptor, interferes with viral protease activity (Mpro), and reduces inflammatory cytokines (TNF-α, IL-6).	(416, 436)
Dengue virus	Reduces viral replication by inhibiting NS2B-NS3 protease, interferes with viral RNA replication, and decreases viral entry into host cells.	(410, 437)
Hepatitis B virus (HBV)	Inhibits viral replication by downregulating HBsAg and HBx expression, reduces transcriptional activation of viral DNA, and inhibits viral assembly.	(409, 438)
Human papillomavirus (HPV)	Inhibits viral gene expression, interferes with viral entry by downregulating viral oncoproteins E6 and E7, and induces apoptosis in infected cells.	(439, 440)
Chikungunya virus (CHIKV)	Inhibits viral replication by interfering with the viral protease function, blocks entry of virus into host cells, and reduces inflammation.	(414)
Respiratory syncytial virus (RSV)	Disrupts viral attachment and fusion by interfering with F and G protein functions, inhibits viral RNA synthesis, and reduces lung inflammation.	(432)
Enterovirus 71 (EV71)	Inhibits viral replication by disrupting viral RNA polymerase activity, reduces cytopathic effects, and interferes with viral entry and attachment.	(441, 442)
Coxsackievirus B3 (CVB3)	Suppresses viral replication by inhibiting viral protease and RNA polymerase activity, reduces virus-induced cell death, and decreases inflammatory response.	(443, 444)
Ebola virus	Inhibits viral entry into host cells by blocking viral glycoprotein binding to the host receptor, reduces viral replication, and interferes with viral RNA synthesis.	(445–447)
Norovirus	Interferes with viral replication, inhibits viral protease activity, and disrupts viral entry into host cells by targeting capsid proteins.	(448, 449)
Cytomegalovirus (CMV)	Inhibits viral entry, interferes with DNA polymerase activity, disrupts viral replication, and reduces cytomegalovirus-induced inflammation.	(450, 451)

### 13.8 Antiparasitic, anti-insects, and antimalarial activity of curcumin

Curcumin demonstrates significant antiparasitic potential, inhibiting the growth of various parasites in the *in vitro* and *in vivo* studies. These include *Plasmodium falciparum* (452)*, Leishmania major, Leishmania donovani* (453)*, Trichomonas vaginalis* (454), *Entamoeba histolytica* (455)*, Toxoplasma gondii* (456)*, Neospora caninum* (457), and *Giardia lamblia* (458).

When coupled with artemisinin, curcumin exhibits additive efficacy against *P. falciparum* and synergistic antiprotozoal activity. In cases involving the highly virulent *Plasmodium berghei* in mice, curcumin has been shown to improve survival rates (459). The extensive resistance of *Plasmodium* species to standard antimalarial medicines, such as chloroquine, presents a significant challenge in malaria management (330). However, curcumin remains effective against chloroquine-resistant *P. falciparum in vitro* and artemisinin-resistant *Plasmodium chabaudi in vivo* (330).

Martinelli et al. (460) emphasized the potential of curcumin as a foundation for innovative malaria treatments. Furthermore, da Silvaa et al. (461) synthesized curcumin monocarbonyl derivatives, which demonstrated superior efficacy against *Trichomonas vaginalis*— a causative agent of for trichomoniasis—when compared to metronidazole (461). These derivatives (3a, 3e, and 5e) exhibited enhanced chemical stability and more significant anti-trichomoniasis activity than natural curcumin (332).

Curcumin’s synergistic potential extends to combination with existing drugs. For example, combining metronidazole with curcumin has shown promise against leishmaniasis, while netilmicin paired with curcumin effectively treats amoebiasis (455, 462). In addition, curcumin displays anthelmintic activity against *Ascaridia galli* and the cestode *Raillietina cesticillus* (463, 464). In malaria treatment, curcumin not only offers direct therapeutic benefits but also enhances the efficacy of existing antimalarial drugs (465).

Recent advancements highlight the use of curcumin in nanoparticle-based drug delivery. Busari et al. (466) demonstrated that curcumin encapsulated in poly(lactic-co-glycolic) nanoparticles exhibits superior antimalarial activity compared to free curcumin (465). Lower doses of the nanoparticle-based formulation showed enhanced efficacy, with *in vivo* toxicity studies confirming its safety at the tested levels (466).

Moreover, Novaes et al. (465) explored the role of curcumin as an adjuvant in benznidazole-based chemotherapy for *Trypanosoma cruzi* infections. Their findings revealed that curcumin enhances the antiparasitic effects of benznidazole while mitigating its side effects, making the combination a promising therapeutic option for Chagas’ disease triggered by *T. cruzi*.

Moreover, Kausar et al. (467) highlighted the significant clinical implications of curcumin derivatives by demonstrating their potential as novel insecticidal agents. Initial computational studies (molecular docking) investigated how curcumin derivatives bind to the *Helicoverpa armigera* Sterol Carrier Protein-2 (HaSCP-2). These simulations revealed that the derivatives predominantly form hydrophobic interactions with key residues in the active site: Phe53, Phe110, and Phe89 (467).

To validate these computational findings, laboratory experiments using fluorescence binding and displacement assays were conducted to determine the actual binding affinities of the curcumin derivatives. Among those tested, Cur10 demonstrated the strongest binding, exhibiting the lowest IC_50_ value of 9.64 μM, while Cur07 and Cur06 showed IC_50_ values of 19.86 μM and 20.79 μM, respectively (467). Crucially, a strong inverse correlation was observed between the ability of the curcumin derivatives to displace a fluorescent probe from HaSCP-2’s sterol binding site and their capacity to inhibit *Sf9* insect cell growth in culture. This finding is clinically important because it supports a novel mechanism of action. These curcumin derivatives likely exert their insecticidal effects by blocking sterol uptake in insects (467).

Since insects, unlike humans and other mammals, cannot synthesize sterols and must obtain them from their diet, inhibiting sterol uptake represents a promising, species-specific insecticidal strategy with potentially low toxicity to non-target organisms. This provides a foundation for developing environmentally friendly and safe pest control alternatives to conventional insecticides (467). This persuasive data underscores curcumin’s significance in antiparasitic and antimalarial therapeutics, facilitating the development of novel treatments aimed at resistant strains and improving the efficacy of current medications. The antiparasitic properties of curcumin are shown in **Table 6**.

**Table 6:** Antiparasitic and antimalarial activity of curcumin.

Parasites	Mechanisms of action	References
*Plasmodium falciparum*	Inhibits hemozoin formation, generates reactive oxygen species, interferes with calcium homeostasis, inhibits trophozoite maturation, and modulates parasite apoptosis pathways.	(468)
*Plasmodium berghei*	Disrupts mitochondrial membrane potential, increases reactive oxygen species production, inhibits parasite growth in the liver and blood stages, and enhances the immune response in host cells.	(469, 470)
*Leishmania donovani*	Induces apoptosis-like cell death by increasing reactive oxygen species, depolarizing the mitochondrial membrane, inhibiting topoisomerase I, and reducing parasite infectivity.	(471)
*Leishmania major*	Triggers apoptosis via caspase activation, increases oxidative stress, and inhibits parasite growth by targeting key enzymes involved in cellular metabolism.	(472)
*Trypanosoma brucei*	Inhibits parasite proliferation by disrupting energy metabolism, increases oxidative stress, induces apoptosis through caspase activation, and inhibits topoisomerase II.	(473)
*Trypanosoma cruzi*	Enhances reactive oxygen species generation, inhibits mitochondrial respiration, induces apoptosis-like death in epimastigotes and amastigotes, and disrupts cell membrane integrity.	(465)
*Toxoplasma gondii*	Inhibits parasite invasion and replication, induces reactive oxygen species-mediated apoptosis, disrupts calcium signaling, and inhibits cyst formation in host tissues.	(474)
*Giardia lamblia*	Induces oxidative stress, inhibits cysteine protease activity, and disrupts the parasite's microtubule network, reducing motility and attachment to host cells.	(458)
*Entamoeba histolytica*	Inhibits cell proliferation, increases reactive oxygen species production, and induces apoptosis-like death, disrupts key enzymes involved in the parasite’s energy metabolism.	(455, 475)
*Schistosoma mansoni*	Inhibits egg production and reduces granuloma size, enhances oxidative stress, disrupts key enzymes, and induces apoptosis in schistosomula and adult worms.	(476)
*Cryptosporidium parvum*	Inhibits parasite replication and oocyst formation, induces oxidative stress in oocysts and sporozoites, and disrupts intracellular growth and invasion.	(477)
*Trichomonas vaginalis*	Induces apoptosis via reactive oxygen species generation, inhibits parasite growth and motility, and disrupts protein synthesis and energy metabolism in trophozoites.	(454)
*Brugia malayi*	Inhibits larval development, induces reactive oxygen species-mediated apoptosis, disrupts mitochondrial function, and blocks key enzymes involved in parasite reproduction.	(478)
*Onchocerca volvulus*	Induces oxidative stress, disrupts mitochondrial integrity, inhibits embryogenesis, and triggers programmed cell death in adult worms and microfilariae.	(479)

### 13.9 Anti-diabetic activity of curcumin

Curcumin, known for its inflammatory and blood sugar-regulating properties, shows promise in preventing and managing type 2 diabetes. A study involving 240 individuals with prediabetes over nine months revealed that curcumin supplementation significantly reduced their risk of developing diabetes (480). While research is still ongoing, most findings thus far are based on animal studies rather than human trials (338). Curcumin’s hypoglycemic, hypolipidemic, antioxidative, and anti-inflammatory effects have been extensively documented in animal models, highlighting its therapeutic potential in diabetes management (339).

Curcumin may enhance insulin sensitivity through multiple mechanisms: (1) improved glucose homeostasis: curcumin may increase hepatic glucokinase activity, facilitating better glucose regulation; (2) reduced hypertriglyceridemia: by boosting lipoprotein lipase activity, curcumin can lower very-low-density lipoproteins (VLDL) and triglycerides, addressing lipid imbalances (481); and (3) enhanced glucose uptake: it promotes the expression of glucose transporter-4 (GLUT4), improving peripheral glucose absorption (482). According to Kim et al. (483) the ability of curcumin to reduce glucose levels may be attributed to its ability to decrease the production of hepatic glucose in the liver by inhibiting gluconeogenesis.

Moreover, curcumin has been proposed to mitigate the vascular complications of diabetes, including diabetic retinopathy, cardiomyopathy, and diabetic nephropathy (484, 485). These findings highlight curcumin’s potential as a multifaceted agent in diabetes prevention and treatment.

### 13.10 Anti-cholesterol activity of curcumin

A study conducted in India demonstrated that curcumin has significant anti-cholesterol properties (346). In the study, ten participants consumed 50 mg of curcumin daily for seven days. This resulted in a 29% increase in high-density lipoprotein (HDL) levels and a 6.11% reduction in blood cholesterol levels. These findings suggested that turmeric may help prevent cardiovascular and vascular disorders in humans (486).

### 13.11 Anti-cancer activity of curcumin

Carcinogenesis involves a complex series of steps, activating multiple metabolic pathways and mediators (487–490). Key molecules such as proliferative enhancers, cytokines, transcription factors, growth factors, apoptosis inhibitors, and growth factor receptors play crucial roles in cancer development (349, 350). Research indicates that curcumin can target these molecules, influencing cancer progression by regulating cell growth and inducing apoptosis (programmed cell death; PCD) (491). Curcumin has also been found to counteract the carcinogenic effects of tobacco condensates (492).

Curcumin suppresses cancer growth and invasion while promoting apoptosis through various cellular signaling pathways (344). By targeting these pathways, curcumin may inhibit tumor development and angiogenesis (354). Although clinical studies highlight its potential anticancer effects, results vary, with some showing significant benefits while others were inconclusive. This variability may be attributed to curcumin’s limited bioavailability, as it is rapidly metabolized and excreted from the body (493, 494). Current research focuses on developing enhanced formulations and delivery methods to increase therapeutic efficacy. The anti-cancer properties of curcumin are shown in **Table 7**.

**Table 7:** Anti-cancer activity of curcumin.

Cancer cell name	Mechanisms of action	References
Breast cancer (MCF-7, MDA-MB-231)	Induces apoptosis via the mitochondrial pathway, downregulates NF-κB and STAT3 signaling, inhibits PI3K/AKT/mTOR pathway, and reduces cell migration and invasion.	(495, 496)
Colon cancer (HCT116, HT29, SW480)	Triggers reactive oxygen species production, induces apoptosis via caspase-3 activation, suppresses Wnt/β-catenin signaling, inhibits COX-2 expression, and blocks tumor progression.	(497, 498)
Prostate cancer (PC-3, DU145, LNCaP)	Inhibits androgen receptor (AR) signaling, induces apoptosis through caspase activation, downregulates NF-κB and AKT pathways, and reduces proliferation and metastasis.	(499)
Lung cancer (A549, H1299)	Suppresses EGFR signaling, induces apoptosis via caspase-3 activation, inhibits cancer cell proliferation, migration, and invasion, and downregulates matrix metalloproteinases.	(500, 501)
Ovarian cancer (SKOV-3, OVCAR-3)	Induces cell cycle arrest at the G2/M phase, promotes apoptosis through the mitochondrial pathway, inhibits NF-κB, and enhances chemosensitivity to cisplatin.	(502)
Pancreatic cancer (PANC-1, MiaPaCa-2)	Inhibits NF-κB and STAT3, induces apoptosis via the mitochondrial pathway, reduces tumor cell invasion and migration, and enhances chemosensitivity to gemcitabine.	(503, 504)
Liver cancer (HepG2, SMMC-7721)	Induces apoptosis via reactive oxygen species generation and caspase-3 activation, inhibits PI3K/AKT and ERK/MAPK pathways, and suppresses metastasis.	(228, 505)
Gastric cancer (AGS, MKN45)	Promotes apoptosis by upregulating Bax/Bcl-2 ratio, inhibits NF-κB and COX-2 expression, induces autophagy, and inhibits cell proliferation.	(506)
Cervical cancer (HeLa, SiHa)	Induces apoptosis via the mitochondrial pathway, inhibits HPV E6/E7 oncogenes, suppresses NF-κB and AKT signaling, and reduces cancer cell migration.	(507, 508)
Leukemia (K562, HL-60, THP-1)	Induces apoptosis through reactive oxygen species generation and caspase activation, blocks cell cycle progression, downregulates NF-κB, and inhibits cancer cell proliferation.	(502)
Glioblastoma (U87, U251, LN229)	Inhibits cancer cell proliferation and migration, induces apoptosis via the mitochondrial pathway, blocks PI3K/AKT/mTOR signaling, and promotes cell cycle arrest at the G2/M phase.	(509, 510)
Bladder cancer (T24, 5637)	Induces apoptosis via activation of caspases, inhibits NF-κB and STAT3 signaling, reduces cell proliferation, migration, and invasion, and enhances sensitivity to cisplatin.	(511)
Melanoma (A375, SK-MEL-28)	Inhibits cancer cell proliferation, migration, and invasion, induces apoptosis via reactive oxygen species generation and caspase activation, and suppresses NF-κB and PI3K/AKT signaling.	(512)
Esophageal cancer (KYSE-150, Eca-109)	Induces apoptosis by upregulating Bax and downregulating Bcl-2, inhibits PI3K/AKT signaling, suppresses cancer cell proliferation, and enhances radiosensitivity.	(513)
Head and neck cancer (FaDu, SCC-4)	Induces cell cycle arrest and apoptosis through caspase activation, inhibits NF-κB signaling, reduces cancer cell migration, and enhances sensitivity to radiation.	(514)
Renal cancer (Caki-1, 786-O)	Induces apoptosis via the mitochondrial pathway, inhibits NF-κB and PI3K/AKT signaling, reduces cancer cell proliferation, and enhances chemosensitivity to sunitinib.	(515)

#### 13.11.1 Breast cancer

Breast cancer, the most prevalent malignancy in women, is heavily influenced by estrogen and its receptors (ER-α and ER-β). Approximately two-thirds of breast cancer cases involve overexpression of these receptors, making them critical therapeutic agents (516). Shao et al. (517) revealed that curcumin exhibits potent anti-invasive properties in estrogen-negative MCF-7 breast cancer cell lines. In estrogen-positive lines, curcumin’s antiproliferative effects are estrogen-dependent. These actions involve upregulation of TIMP-1 (tissue inhibitor of metalloproteinase) and MMP-2 (matrix metalloproteinase), both key factors in tumor metastasis (517).

Further research by Calaf et al. (518) demonstrated that curcumin disrupts microtubule formation and cell division checkpoints, inducing apoptosis and inhibiting cell proliferation in MCF-7 cells. When combined with paclitaxell, curcumin enhanced apoptotic activity more effectively than either agent alone (377).

Several analogs have been developed and tested to improve curcumin’s anticancer efficacy. Two promising analogs, PAC and EAC, showed superior blood stability, water solubility, bioavailability, and distribution compared to curcumin. Remarkably, these analogs were five times more effective in inducing apoptosis in breast cancer-causing toxicity (519).

#### 13.11.2 Lung cancer

Lung cancer is among the most lethal types of cancer worldwide, contributing significantly to morbidity and mortality rates (520). Non-small cell lung cancer (NSCLC) represents approximately 85% of all lung cancer cases. Unfortunately, two-thirds of NSCLC cases are diagnosed at advanced stages, making treatment challenging due to drug resistance (521). This highlights the urgent need for innovative adjunctive chemotherapy strategies to enhance current treatments, mitigate side effects, and reduce toxicity without compromising efficacy (380).

Curcumin has emerged as a promising candidate for this purpose. Several studies have demonstrated its ability to inhibit NF-κB activation, a key factor in promoting carcinogen-induced processes such as apoptosis suppression, cellular transformation, invasion, metastasis, chemoresistance, and inflammation (522, 523). A novel curcumin analog, JZ534, has shown enhanced anticancer properties in lung cancer cell lines (524). This compound effectively inhibited cell proliferation, induced PCD, and elevated apoptosis-related proteins such as caspase 3, Bax, and p53. Remarkably, JZ534 exhibited greater anticancer efficacy than curcumin at equivalent doses (410).

#### 13.11.3 Cervical cancer

Curcumin has also shown significant potential against cervical cancer, particularly due to its antimetastatic properties. Research indicates that curcumin inhibits the migration and invasion of cancer cells by suppressing matrix metalloproteinases (MMP-2) and (MMP-9), which are enzymes that facilitate cancer spread by degrading the extracellular matrix (525, 526). In addition, curcumin effectively suppresses telomerase activity, a critical factor in cervical cancer progression, making this mechanism one of its most potent anticancer effects in this context (527).

A novel curcumin analog, EF24, has demonstrated superior anticancer potential. According to Adams et al. (528), EF24 exhibits enhanced bioavailability and robust biological effects compared to curcumin. Furthermore, studies by Tan et al. (529) suggest that EF24 is 10–20 times more effective than curcumin in treating cervical cancer. This improved efficacy indicates the potential of EF24 as a powerful therapeutic option for combating cervical cancer (415).

#### 13.11.4 Prostate cancer

Prostate cancer responds well to anti-androgen treatment when detected early. However, as the disease progresses, cancer cells often develops resistance to hormone deprivation therapy, leading to castration-resistant prostate cancer (CRPC) (530). In a recent clinical trial, more than half of CRPC patients showed a prostate-specific antigen (PSA) response when treated with a combination of curcumin and docetaxel. Remarkably, 88% of responders exhibited a PSA response within the first three treatment cycles (531).

The novel curcumin analogs, RL118 and RL121 on PC3 and DU145 cell lines demonstrated a potent cytotoxic effect on CRPC. These analogs induced apoptosis, suppressed nuclear factor NF-_k_B activity and arrested cells in the G2/M phase of the cell cycle (532).

#### 13.11.5 Pancreatic cancer

Pancreatic cancer is one of the leading causes of cancer-related deaths globally, responsible for approximately 7% of all such fatalities. Unfortunately, this cancer shows limited responsiveness to radiation and chemotherapy treatments (533). As a result, alternative therapies, including the use of phytochemicals, have been explored. *In vitro* studies revealed that difluorinated-curcumin (CDF), a curcumin derivative, effectively inhibits the growth and survival of pancreatic cancer cells across various cell lines (534). Another derivative, GO-Y030, exhibited stronger inhibitory effects on pancreatic cell lines than curcumin, likely through the suppression of STAT3 signaling pathways (535).

#### 13.11.6 Colorectal cancer

Colorectal cancer ranks as the fourth most diagnosed malignancy in high-income countries and the fifth in low-income regions. Chemo-preventive strategies have been developed to slow or halt carcinogenesis (536). Since 1995, numerous studies have demonstrated curcumin’s ability to inhibit and reduce the proliferation of colorectal cancer cells (537, 538).

### 13.12 Anticoagulant activity of curcumin

Turmeric, primarily due to its active compound curcumin, has been extensively studied for its anticoagulant properties. Curcumin inhibits blood clotting by interacting with coagulation factors and platelets. Unlike traditional anticoagulant drugs like warfarin, curcumin demonstrates fewer adverse effects, making it a promising natural alternative (425, 426).

Soni and Salh (539) revealed that curcumin effectively inhibits platelet aggregation and prevents blood clot formation. Similarly, Palathy et al. (540) found that curcumin enhances fibrinolytic factor activity while reducing coagulation factor activity facilitating clot breakdown. These findings suggest that *C. longa* may possess anticoagulant properties beneficial for individuals at risk of cardiovascular diseases or thrombosis (426).

While curcumin holds potential as a therapeutic anticoagulant, further studies are essential to elucidate its mechanisms and optimize its clinical applications fully.

### 13.13 Antinociceptive activity of curcumin

Curcumin has shown significant antinociceptive effects in preclinical studies, particularly for neuropathic and inflammatory pain. A study by Zhu et al. (541) demonstrated that curcumin alleviates postoperative pain in rats and accelerates recovery. However, preoperative administration of curcumin did not influence the postoperative pain threshold or recovery rates (542).

Further research evaluated the antinociceptive effects of curcumin encapsulated in PLGA-based curcumin administered intravenously or intrathecally in mice. Intravenous PLGA-based curcumin effectively reduced pain response in formalin and zymosan-induced hyperalgesia models. Intrathecal administration of low doses significantly mitigated allodynia caused by sciatic nerve ligation, while high doses provided prolonged antinociceptive effects (454).

In contrast, pure curcumin administered intrathecally induced only short-term, strong pain relief at high doses. The enhanced efficacy of PLGA-based curcumin is attributed to reduced production of cytokines and brain-derived neurotrophic factor (BDNF) in the spinal cord, as observed in neuropathic pain models (454). This study highlights PLGA-based curcumin potential as an innovative approach to pain management and underscores the therapeutic promise of curcumin nano-formulation (543).

### 13.14 The anti-Alzheimer’s activity of curcumin

Alzheimer’s disease (AD), a progressive neurodegenerative condition, remains without a definitive cure despite extensive research. Its complex etiology and pathophysiology suggest that the disease arises from multiple factors rather than a single cause (455). A hallmark feature of AD is the aggregation of extracellular amyloid plaques. Additionally, oxidative damage, driven by ROS and biometals like iron, plays a significant role in the disease’s progression (544).

Given the multifaceted nature of AD, there is an urgent need for therapeutic agents capable of targeting multiple pathological pathways (545). Curcumin has emerged as a promising candidate due to its potent anti-inflammatory and antioxidant properties (546). Chen et al. (545) developed a series of curcumin-based compounds and evaluated their potential for treating AD. These compounds demonstrated enhanced inhibitory activity compared to curcumin itself. They also outperformed the reference antioxidant Trolox, exhibiting superior metal-chelating abilities (iron and copper), antioxidant properties, and a capacity to mitigate metal-induced amyloid aggregation (456). Among the tested derivatives, a compound known as A4 showed the most promising results, surpassing other curcumin derivatives in efficacy (456). These findings highlight the potential of A4 as a lead compound for developing multifunctional anti-Alzheimer drugs and highlight the importance of further structural optimization to enhance its therapeutic effects (545).

Moreover, Lan et al. (547) revealed a crucial clinical application of curcumin in mitigating cerebral ischemia-reperfusion (I/R) injury, a significant cause of neuronal damage and neurological dysfunction. The findings indicate that cerebral I/R injury not only causes a specific type of programmed neuronal death called PANoptosis but also triggers microglia to adopt a pro-inflammatory (M1) phenotype, both in living organisms and *in vitro*. The research demonstrates that pretreatment with curcumin significantly enhanced the proliferative capacity and anti-inflammatory potential of olfactory mucosa-derived mesenchymal stem cells (OM-MSCs). The “curcumin-primed” OM-MSC (CUR-OM-MSC) group exhibited a more pronounced reduction in PANoptotic neuronal death and showed better recovery of neurological function compared to the group treated with OM-MSCs alone (547).

The bioinformatic analysis provided a key mechanistic insight: microRNA-423–5p (miRNA-423–5p) expression was notably upregulated in CUR-OM-MSCs compared to unprimed OM-MSCs (547). This suggests that CUR-OM-MSC treatment induces a beneficial shift of microglia to an anti-inflammatory (M2) phenotype by releasing miRNA-423–5p. This miRNA-423–5p targets nucleotide-binding oligomerization domain 2 (NOD2), which is an upstream regulator of the NF-kappaβ and Mitogen-Activated Protein Kinase (MAPK) signaling pathways. By modulating these pathways, the intervention attenuates PANoptotic neuronal death resulting from cerebral I/R injury (547).

The clinical importance of these findings is substantial. They provide the first demonstration of PANoptotic neuronal death in cerebral I/R conditions and highlight a novel mechanism by which curcumin-primed stem cells can reduce neuroinflammation and improve outcomes (547). This combined approach of curcumin and OM-MSCs offers a promising and potentially efficacious therapeutic strategy for ischemic stroke, addressing multiple facets of injury, including inflammation and specific forms of cell death (547). Curcumin itself has been shown to offer neuroprotection in cerebral ischemia through various mechanisms, including anti-oxidation, anti-inflammation, anti-apoptosis, and protection of the blood-brain barrier, making this combined strategy particularly compelling (547).

## 14 Other applications of curcumin

### 14.1 Enhancing skin glow

Numerous physiological and pharmacological processes are carried out by curcumin, known for its physiological and pharmacological properties, including its role in skin health (548). Turmeric extract containing curcuminoids is widely used in topical and oral skincare products. It has been suggested to combat signs of aging skin caused by sun exposure, injuries, increased skin thickness, and reduced elasticity. However, these claims are supported by limited experimental evidence (549).

Sebum, a crucial secretion produced by sebaceous glands, plays a key role in protecting the skin against harmful chemicals and pathogens. Sebum, which constitutes about 95% of skin, includes triglycerides, free fatty acids, waxes, squalene, sterols, and glycophospholipids. By retaining moisture, it enhances the skin’s emollient function and overall resilience (550).

**Figure 8:**
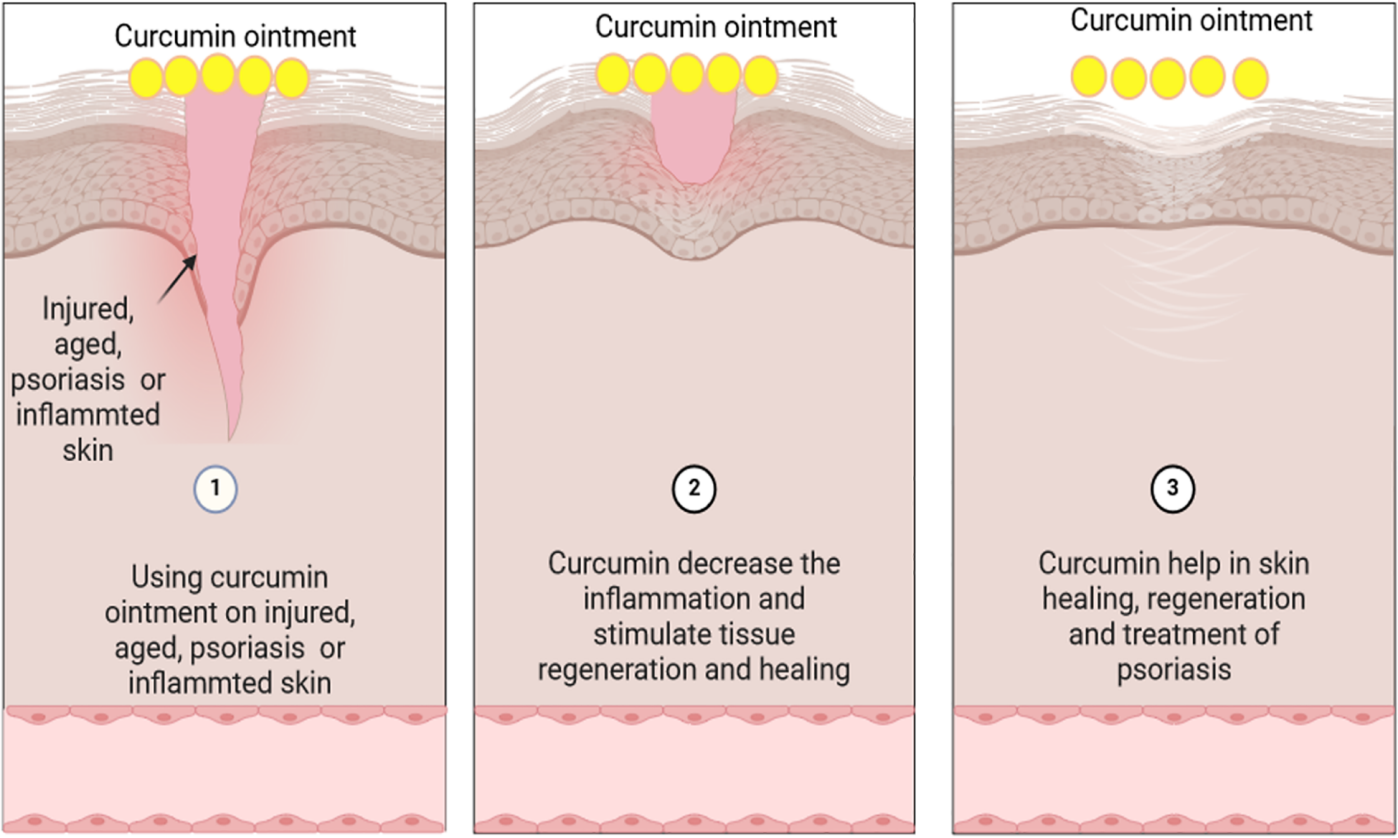
Effect of curcumin on skin lesions.

The effect that curcumin has on skin lesions is illustrated in **Figure 8**.

### 14.2 Psoriasis treatment

Psoriasis, a chronic inflammatory disease, primarily affects the skin and sometimes the joints, bones, tendons, and nails. The most common form, psoriasis vulgaris, is characterized by oval lesions with white-silver scales, typically found symmetrically on the scalp, lower back, elbows, and knees (551). Recent studies highlight curcumin’s potential in managing psoriasis due to its antioxidant and anti-inflammatory properties (552).

Research suggests curcumin reduces oxidative stress in psoriatic lesions and inhibits elevated phosphorylase kinases, a key factor in psoriasis pathology (553). *In vitro* studies using doses of 25 and 50 µM showed that curcumin suppressed the growth of psoriatic-like cells (HaCaT cells) by reducing the production of proinflammatory cytokines such as interleukin 17, tumor necrosis factor-α (TNF-α), interferon-γ, and interleukin-6. In addition, curcumin improves skin barrier function by upregulating involucrin (iNV) and filaggrin (FLG) proteins, which are critical for maintaining healthy skin (554).

### 14.3 Scabies treatment

Traditional Indian medicine systems, including Ayurvedic and Sidha, have long utilized *Azadirachta indica* (neem) and *C. longa* (turmeric) for treating chronic ulcers and scabies (465). In a large-scale study, a paste made from neem and turmeric was applied to 814 individuals with scabies (465). Remarkably, 97% of cases were cured within 3–15 days. This method offers cost-effective, accessible, highly effective treatment, particularly for rural communities in resource-limited settings. Furthermore, no adverse reactions or toxicity were observed during the treatment (555).

### 14.4 Depression management

Curcumin has garnered significant attention in recent years for its potential role in addressing the underlying mechanisms of depression (466). Research suggests that curcumin interacts with various systems implicated in the pathophysiology of major depressive disorder (MDD). It appears to regulate neurotransmitter levels, inflammatory pathways, neuroplasticity, excitotoxicity, and disturbances in the hypothalamus-pituitary-adrenal (HPA) axis (466). In addition, curcumin mitigates oxidative and nitrosative stress, insulin resistance, and imbalances in the endocannabinoid system, making it a promising candidate for managing MDD (556).

### 14.5 Reducing asthma attacks

As a chronic inflammatory disease of the airways, bronchial asthma remains challenging to treat, with no current therapies altering its progression (467). However, curcumin’s potent anti-inflammatory properties have shown promise in mitigating symptoms. Both *in vitro* and *in vivo* studies highlight curcumin’s pharmacological potential to reduce airway inflammation, making it a valuable adjunct in managing bronchial asthma (557).

### 14.6 Treatment for irritable bowel syndrome

Irritable bowel syndrome (IBS), a prevalent gut-brain axis disorder, is characterized by abdominal pain, discomfort, and altered bowel habits without anatomical abnormalities (480). Despite its global impact, the pathophysiology of IBS remains unclear, and treatment primarily involves supportive therapies (480). Recent studies in animals and humans suggest that curcumin may offer therapeutic benefits for IBS, alleviating symptoms and improving quality of life through its anti-inflammatory and gut-modulating properties (558).

## 15 Conclusion

This review highlights turmeric’s (*C. longa*) medicinal potential, primarily due to its active compound, curcumin. While clinical trials confirm curcumin’s safety and efficacy at appropriate dosages for various human and animal diseases, its clinical utility is hindered by poor bioavailability due to limited absorption and rapid metabolism. To overcome these challenges, strategies that combine curcumin with complementary components are being explored, thereby enhancing its therapeutic potential. Emerging research highlights curcumin’s diverse biological activities and its potential as a therapeutic agent for various conditions, provided optimal dosages are achieved. Curcumin demonstrates significant therapeutic promise across a range of conditions, but its poor bioavailability remains a critical challenge. The current review suggests that ongoing efforts to combine curcumin with complementary components are crucial for enhancing its therapeutic potential.

Future research should prioritize the development of novel delivery systems to enhance curcumin’s absorption and stability, as well as further explore synergistic combinations with other compounds. Additionally, more in-depth studies are needed to fully elucidate curcumin’s diverse mechanisms of action, particularly in specific disease contexts. Rigorous and comprehensive clinical trials are essential to establish optimal dosages, assess long-term safety profiles across diverse patient populations, and confirm their efficacy in treating various human diseases. These comprehensive investigations will be crucial for unlocking the full potential of curcumin as an effective and widely applicable therapeutic agent.
